# Effect of the Activation Agent on Carbons Derived from Exhausted Olive Pomace as Sulfur Hosts in Sustainable Lithium–Sulfur Batteries

**DOI:** 10.1021/acsami.5c12218

**Published:** 2025-08-25

**Authors:** Hansi Martínez-Alvarenga, Azahara Cardoso-Almoguera, María del Carmen Gutiérrez, Almudena Benítez, María de los Angeles Martín, Alvaro Caballero

**Affiliations:** † Departamento de Química Inorgánica e Ingeniería Química, Instituto Químico para la Energía y el Medioambiente (IQUEMA), 83138 Universidad de Córdoba, 14071, Córdoba, Spain; ‡ Campus de Excelencia Internacional Agroalimentario ceiA3, Universidad de Córdoba, Campus Universitario de Rabanales, N-IV, km 396, Córdoba, 14071, Spain

**Keywords:** olive oil waste, chemical activations, porous carbons, sustainable sulfur-cathode, Li−S batteries

## Abstract

*Alpeorujo*, a highly abundant byproduct of olive oil extraction, poses serious environmental concerns due to its massive accumulation. In parallel, the urgent global transition toward greener, more efficient, and sustainable energy storage technologies remains a critical challenge. This work addresses both issues simultaneously by pioneering the valorisation of *alpeorujo* through its transformation into activated carbons (ACs) for application as cathode matrices in lithium–sulfur (Li–S) batteries. A straightforward, one-step calcination route, aligned with green chemistry principles, was employed using KOH, ZnCl_2_, and H_3_PO_4_ as chemical activating agents (AA). The resulting ACs, with a remarkable high carbon content above 85%, exhibited diverse and well-developed porosities and were simply combined with sulfur (AC@S) via mechanical grinding, enabling 70% sulfur infiltration into the porous carbon network. The ACs displayed distinctive textural properties depending on the AA used. Notably, all ACs contained considerable amounts of nitrogen, acting as a self-doped heteroatom that enhances electrochemical functionality without additional treatments. Electrochemical testing revealed excellent performance, particularly for the AC obtained from H_3_PO_4_, which delivered specific capacities of 1100 mAh/g. Near-ideal Coulombic efficiency (∼100%) and a significantly lower decay rate (around 0.07%/cycle) were maintained over ultralong-term cycling. Furthermore, all ACs demonstrated strong polysulfide adsorption, with the AC obtained from H_3_PO_4_ achieving the best results, thereby confirming its outstanding electrochemical behavior. These results underscore the potential of *alpeorujo* as a sustainable precursor for high-performance carbon materials, bridging waste valorisation and green energy storage solutions.

## Graphical abstract

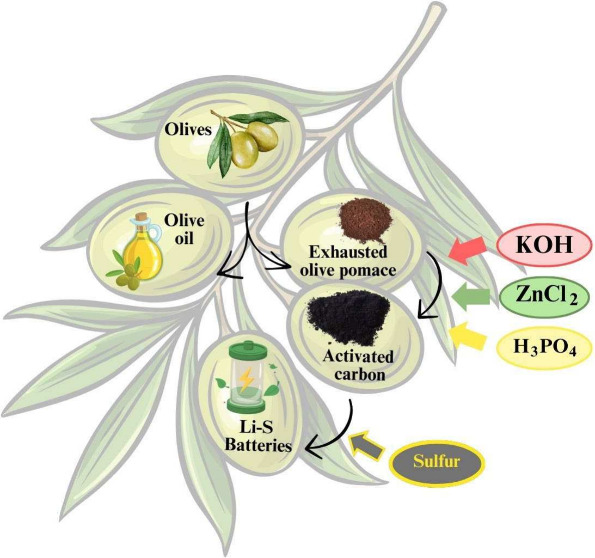

## 1 Introduction

One of the most significant global issues is the overwhelming amount of waste generated by industrial development, particularly from the agri-food industry. While not representing the primary or most hazardous, these residues have encouraged interest in exploring new utilization and valorisation alternatives to mitigate the environmental impacts they generate.^1^ Additionally, there is a growing global need to develop more efficient energy systems due to the problems caused by global warming, greenhouse gas emissions, and the depletion of fossil fuels.^2^ As a solution, the development of batteries capable of storing the energy generated by renewable sources during operational periods is proposed, ensuring supply during times when they are not available.^3^ It is also important to emphasize the need for batteries as a fundamental component in next-generation electric mobility, aimed at reducing dependence on oil and other fossil fuel energy sources.

Regarding waste generation, the olive oil production industry ranks among the most traditional and significant food industries in Mediterranean countries, with Spain being the world’s leading country in terms of both acreage and production.^4^ However, one of the major issues with olive oil extraction is the huge amount of waste it produces (exhausted olive pomace, commonly known as *alpeorujo* or *alperujo*). Approximately 80% of the material generated during olive processing is waste, with the average oil content being around 20 wt %.^5^ Over decades, this has led to massive accumulation of these residues, causing high environmental pollution. Consequently, seeking solutions for their proper management, treatment, and valorisation is necessary. One cost-effective option for utilizing *alpeorujo* is through soil application as an organic amendment obtained after composting, providing a substantial amount of potassium. However, this waste, due to its acidic pH and high salinity, could negatively impact soil properties and plant development. Another alternative is cocomposting *alpeorujo* with other cosubstrates to create compost with suitable characteristics that comply with fertilizer regulations.^6^

Continuing with the valorisation processes, authors such as A. Messineo et al.^7^ evaluated the biogas recovery from *alpeorujo* through anaerobic digestion, achieving a conversion rate of up to 89%. Another way to valorise *alpeorujo* is through the extraction of high-value bioactive compounds. In this regard, several phenolic compounds with antioxidant potential have been identified in *alpeorujo* biomass.^8^ Finally, an interesting valorisation alternative for *alpeorujo* is its transformation into ACs.^9,10^ In this context, the synthesis of these carbons from organic waste has caused growing interest within the scientific community due to its simple, eco-friendly, and cost-effective preparation. Apart from the use of biomass-derived ACs in applications such as catalysis^11^ and adsorption,^12^ their utilization as electrode materials for energy storage supercapacitors has been previously reported.^13^

Additionally, in recent years, the use of renewable and environmentally sustainable cathode materials for Li–S batteries has been extensively explored. This technology has attracted significant attention in the energy storage sector due to its elevated theoretical capacity (1672 mAh/g), high theoretical energy density (2600 Wh/kg), and economic advantages compared to lithium-ion batteries.^14^ Previous studies have demonstrated the utilization of biomass-based waste to produce ACs with appropriate properties to act as conductive matrices for Li–S batteries.^15^ However, no bibliographic evidence has been found on the use of *alpeorujo* in this battery technology. Regarding waste from the olive industry, only ACs derived from olive stones have been studied for Li–S batteries. Moreno et al.^16^ evaluated the electrochemical performance of a Li–S cell fabricated with AC from olive stones. In this case, this AC was synthesized through physical activation under a steam stream and pyrolysis at 700 °C, obtaining a material with 92% C and a specific surface area of 600 m^2^/g. The prepared cell exhibited a capacity of approximately 670 mAh/g, with notable capacity retention over 100 cycles. However, the olive stones represent only a minimal fraction of the *alpeorujo*, which is the major and most problematic residual byproduct of the olive oil industry. This work employs physical activation for the synthesis of the AC, a method significantly more complex compared to chemical activation. One of the main disadvantages of physical activation is the long processing time required, which, in turn, leads to high energy consumption and a significant increase in the final production costs. On the other hand, chemical activation is more cost-effective, as it requires shorter processing times and offers higher efficiency in carbon production.^17^ Moreover, ACs produced through chemical activation exhibit a more porous structure compared to those obtained by physical activation, making them more effective for different applications.^18^ Chemical agents such as KOH, ZnCl_2_, and H_3_PO_4_ have been studied in the synthesis of ACs from various biomass residues as cathodes for Li–S batteries, such as corncob, peanut shell and pistachio shell.^15^ These agents promote the formation of controlled and hierarchical porosities, effectively confining sulfur and mitigating the harmful shuttle effect caused by the formation of lithium polysulfides (LiPSs). However, most studies focus on a single activating agent (AA), varying other synthesis parameters of biomass-derived ACs, without exploring the impact of alternative chemical agents.

In this context, this work introduced a novel approach by exploring *alpeorujo* as a material for Li–S batteries, an application that, to the best of our knowledge, has not been previously investigated. The strategy focused on preparing highly porous carbons through chemical activation with agents of varying acid–base characteristics (KOH, ZnCl_2_, or H_3_PO_4_), and one-step pyrolysis. This simple transformation process, using different activating agents, has enabled the production of materials with variable textural properties and nitrogen doping in diverse proportions. In particular, the use of H_3_PO_4_ as an activating agent not only promotes nitrogen incorporation into the carbon structure but also introduces phosphorus, which significantly enhances the material’s electronic conductivity. This synergy between nitrogen and phosphorus self-doping has a beneficial effect on sulfur nucleation, provides efficient polysulfide adsorption, and shows a substantial increase in specific capacity. As a result, the developed materials have demonstrated remarkable electrochemical performance, achieving specific capacities up to three times higher than those of conventional commercial batteries, confirming their potential as advanced and sustainable solutions for energy storage in Li–S batteries.

## 2 Material and Methods

### 2.1 Preparation of ACs and Their Sulfur Composites (AC@S)

The *alpeorujo* used in this work was supplied by the Andalusian cooperative society, *″Nuestra Señora de la Consolación″* (Doña Mencía, Córdoba, Spain). Due to its granular texture and elevated moisture content, the *alpeorujo* feedstock was initially dried in the laboratory at 65 °C. Subsequently, it was processed through manual grinding to ensure a uniform particle size distribution. After drying and homogenizing the *alpeorujo*, it was subjected to physicochemical analysis, in accordance with the methodology detailed in our previous work.^19^ The analysis of the aqueous extract (1:25 w/w ratio) included measurements of pH, soluble total carbon (TC, mg/kg), soluble inorganic carbon (IC, mg/kg), soluble total organic carbon (TOC, mg/kg), and soluble total nitrogen (TN, mg/kg). Meanwhile, the solid fraction was analyzed for moisture (%), fixed solids (FS, mg/kg), volatile solids (VS, mg/kg), total Kjeldahl nitrogen (N-TKN, mg/kg), and phosphorus content (P–P_2_O_5_, mg/kg). The elemental composition of the alpeorujo ashes, produced by carbonizing the raw material at 550 °C, was determined using X-ray fluorescence (XRF). The spectra were recorded with a Rigaku ZSX Primus IV wavelength-dispersive XRF (WDXRF) spectrometer.

The preparation process of ACs consisted of a simple synthesis, without long-duration, preactivation steps or biomass precarbonization, based on our previous study,^19^ which also included a detailed evaluation of both condensable and noncondensable gaseous streams produced during the transformation of alpeorujo into AC. In this case, the dried *alpeorujo* was mixed with three different AAs: potassium hydroxide (KOH, Panreac, 85%) or zinc chloride (ZnCl_2_, Sigma-Aldrich, 98%) in solid form, or a solution of phosphoric acid (H_3_PO_4_, Panreac, 85%). In all cases, a 2:1 mass ratio of *alpeorujo*:AA was used to minimize the consumption of chemical agents, promoting both its cost-effectiveness and scalability. One-step pyrolysis was performed in a tubular oven under a nitrogen atmosphere, using a flow rate of 50 mL/min. This stage was carried out with a target temperature of 900 °C and a gradient of 10 °C/min. Finally, to remove residues of AAs and any soluble inorganic compounds, ACs were washed using 6 M HCl (Panreac, 37%) and water until reaching neutral pH. The ACs were denoted as AC, referring to activated carbon, next to the letter K, Zn, or P, according to the AA used (KOH, ZnCl_2_ or H_3_PO_4_). Therefore, the ACs were called AC-K, AC-Zn, and AC-P, respectively.

The preparation of the AC and sulfur composite (AC@S) was carried out through wet milling, which is an easy, scalable, and economical process. In detail, a 1 g mixture was prepared, containing 70 wt % of sulfur (S, VWR Chemicals) and 30 wt % of the synthesized AC. Both substances were placed in a 250 mL agate jar, followed by the addition of 2.5 mL of pure ethanol (Panreac) used as an organic medium to promote a homogeneous mixture, facilitating the infiltration of sulfur into the pores of the carbonaceous matrix. The milling was then performed in a planetary ball mill at 300 rpm for 3 h. Once the grinding process was complete, the obtained mixture was recovered and dried in an oven at 50 °C for 12 h to ensure the total evaporation of the ethanol. The AC@S composites were denoted as AC-K, AC-Zn, or AC-P, based on the specific carbonaceous sample used in their preparation, followed by the suffix @S, indicating the incorporation of sulfur in their composition. Thus, the AC@S composites were designated as AC-K@S, AC-Zn@S, and AC-P@S.

### 2.2 Characterization of the Synthesized Materials (AC and AC@S)

A comprehensive characterization of the synthesized ACs, as well as their corresponding sulfur composites, was conducted to analyze their textural, structural, compositional, and morphological properties. The porosity of the AC samples was assessed using nitrogen adsorption–desorption measurements at – 196 °C (77 K) recorded with a Micromeritics ASAP 2020 M analyzer. From the N_2_ adsorption isotherm, the specific surface area (*S*_BET_) was determined using the Brunauer–Emmett–Teller (BET) equation, with relative pressures ranging from 0.04 to 0.20. The total pore volume (V_T_) was calculated at a relative pressure of P/P_0_ = 0.98. The pore size distribution was assessed using density functional theory (DFT). The t-plot method was employed to determine the micropore area (S_micro_) and micropore volume (V_micro_). X-ray diffraction (XRD) was used to study the structures of both the ACs and the AC@S composites. The diffractograms were recorded using a Bruker D8 Discover X-ray diffractometer, equipped with a LynxEye detector, using Cu K_α_ radiation (λ = 1.5406 Å). Raman spectra were obtained with a Leica microscope with a Renishaw CCD camera detector (578 × 400). The selected spectral range was 1000 – 2000 cm^–1^, and ten scans were collected for each spectrum to improve the signal-to-noise ratio. Thermogravimetric analysis (TGA) was carried out using a Mettler Toledo-TGA/DSC analyzer. The measurements of the AC samples were performed in an oxygen atmosphere (flow rate of 100 mL/min), at a temperature range from 25 to 900 °C and a ramp rate of 10 °C/min. To confirm the presence of N, and specifically, P in the sample activated with H_3_PO_4_, X-ray photoelectron spectroscopy (XPS) measurements were performed using a PHOIBOS HSA3500 150 R6 spectrometer equipped with a monochromatic Al K_α_ source (hν = 1486.7 eV). The measurements of the AC@S composites were conducted in a nitrogen atmosphere (flow rate of 100 mL/min), at a temperature range between 25 and 600 °C and a ramp rate of 5 °C/min. To identify the functional groups present in the ACs, Fourier transform infrared (FTIR) spectral analyses were performed in the 4000 – 400 cm^–1^ range in transmission mode using a PerkinElmer Frontier instrument. The multielement composition of the ACs was determined by wavelength dispersive X-ray fluorescence (WDXRF, Rigaku ZSX Primus IV spectrometer), and the nitrogen content was obtained using an elemental analyzer (EA, LECO CNS928 microanalyser). Finally, to confirm the presence of P, particularly in the AC activated with H_3_PO_4_, and the homogeneity of sulfur incorporation into the AC matrix, scanning electron microscopy (SEM) and energy dispersive X-ray spectroscopy (EDS) was utilized (JEOL JSM-7800F SEM equipped with EDS). This technique provided qualitative information on the composition of the AC@S composites, as well as elemental distribution images through mapping.

### 2.3 Electrode Preparation and Cell Assembly

To synthesize the cathodic electrode, the following components were combined: AC@S as the electroactive material, Super P conductive carbon black (Timcal) as the conductive agent, and polyvinylidene fluoride (PVDF, Sigma-Aldrich) as a binder, in a weight ratio of 80:10:10, respectively. Then, *N*-methyl-2-pyrrolidone was added until a homogeneous slurry was formed. Next, the electrode slurry was uniformly deposited onto the current collector using a blade coating technique (MTI Corp.), ensuring a consistent film thickness across the substrate. Subsequently, the electrodes were dried at 50 °C in an oven and then punched into discs with a diameter of 12.8 mm. Afterward, the electrodes were further dried in a vacuum oven (Buchi B-585) at 45 °C for 3 h to ensure the absence of oxygen and remove any remaining moisture. Following this, they were transferred into an Ar-filled glovebox (M-Braun 150; H_2_O, O_2_ < 0.1 ppm) for the assembly of coin-type cells (CR2032 model). A porous polyethylene membrane with a 25 μm thickness and 16 mm diameter (Celgard 2400) was impregnated with the electrolyte solution and used as a separator. The electrolyte solution was composed of two lithium salts, 1 M LiTFSI and 0.4 M LiNO_3_, dissolved in a mixture of organic solvents: 1,2-dimethoxyethane (DME, Sigma-Aldrich) and 1,3-dioxolane (DOL, Sigma-Aldrich) in a 1:1 v/v ratio. The electrolyte volume was adjusted according to the sulfur loading of the electrode. For electrodes with a sulfur loading of ∼1.5 mg/cm^2^, an electrolyte-to-sulfur (E/S) ratio of 30 μL/mg_S_ was employed. Additionally, tests were carried out at higher sulfur loadings (∼6.0 mg/cm^2^) using a reduced E/S ratio of 11 μL/mg_S_ to evaluate the electrochemical performance under more practical conditions. The fabricated cells were designated with the same names as the composites, but preceded by the prefix Li/, resulting in Li/AC-K@S, Li/AC-Zn@S, and Li/AC-P@S.

### 2.4 Electrochemical Characterization of the Electrodes

Cyclic voltammetry (CV) and electrochemical impedance spectroscopy (EIS) were employed to study the electrochemical process. Both experiments were conducted using an Autolab PGSTAT-204 instrument from Metrohm (Herisau, Switzerland). To determine the Li^+^ diffusion coefficients, CV measurements were performed at variable rates of 0.05, 0.08, 0.1, and 0.2 mV/s within the potential range of 1.5 to 3.0 V vs Li/Li^+^. EIS measurements were carried out at the open-circuit voltage (OCV) after four CV cycles, covering frequencies from 500 kHz to 0.1 Hz with a 10 mV amplitude signal. The ZView 2 software from Scribner Associates was employed to fit the equivalent circuit during the simulation. Galvanostatic cycling tests were conducted using an Arbin BT2143 battery tester (Arbin Instruments, College Station, USA) within a voltage window of 1.7–2.7 V. The tests were carried out at constant current densities of 0.2C, 0.5C, and 1C (where 1C = 1675 mA/g) over 150, 300, and 500 cycles, respectively. Additionally, the electrical conductivity of the electrodes was measured using a standard four-point probe resistivity system (P2010A2, Ossila). For each sample, measurements were taken at five different positions, and the average conductivity was calculated.

### 2.5 Lithium Polysulfide Adsorption Tests

Prior to the lithium polysulfide (LiPS) adsorption tests, 15 mg of each AC sample was vacuum-dried at 110 °C overnight. Moreover, 0.5 M Li_2_S_6_ solution was prepared by reacting Li_2_S and sulfur in a 1:5 molar ratio and dissolving them in a DOL:DME (1:1 v:v ratio) solvent mixture. The resulting solution was stirred at 60 °C for 24 h inside an Ar-filled glovebox, forming a brownish-red LiPS solution. For the LiPS adsorption test, 15 mg of dry AC was dispersed in a 0.5 mM Li_2_S_6_ solution. The mixture was subjected to analysis using a Jasco V-750 double-beam UV/visible spectrophotometer. UV–visible spectra were recorded at 10 min intervals, allowing for continuous monitoring of the absorbance at the maximum wavelength of 420 nm. This systematic approach ensured a precise and reliable measurement of the spectral changes over time. Additionally, XPS measurements were also performed on the carbon samples after polysulfide adsorption (PHOIBOS HSA3500 150 R6 spectrometer equipped with a monochromatic Al K_α_ source).

### 2.6 Nucleation and Dissolution Test of Li_2_S

A solution of Li_2_S_8_ was prepared similarly to Li_2_S_6_ by dissolving sulfur powder and Li_2_S in a molar ratio of 7:1, resulting in a 0.2 M dark brown solution. The mixture was kept under constant stirring at 60 °C for 24 h. The whole preparation process of these Li polysulfides was carried out inside a previously Ar-filled glovebox. For these measurements, CR2032 coin cells with carbon-only electrodes (not including sulfur) in the AC-K, AC-Zn, and AC-P variants were used, with lithium metal as the anode and a polyethylene membrane (Celgard 2400) as the separator. On the anode side, 25 μL of 1 M LiTFSI solution was added, and while 25 μL of 0.2 M Li_2_S_8_ with 1 M LiTFSI solution was added on the cathode side. For the nucleation process, the assembled cells were initially discharged galvanostatically at a constant current of 0.112 mA until a voltage of 2.05 V was reached, then further potentiostatically discharged at a constant potential of 2.01 V until the current was lower than 0.01 mA or the total discharge time reached 30,000 s. For the dissolution test, the above cell was first galvanostatically discharged until 1.7 V at 0.112 mA, and then potentiostatically discharged under a constant potential of 1.7 V for 30 min to ensure the conversion of soluble lithium polysulfides into insoluble Li_2_S. Finally, the cells were potentiostatically charged at 2.4 V for the oxidation of Li_2_S to LiPSs.

Figure 1 presents a schematic overview of the experimental process used for fabricating Li–S batteries.

**Figure 1:**
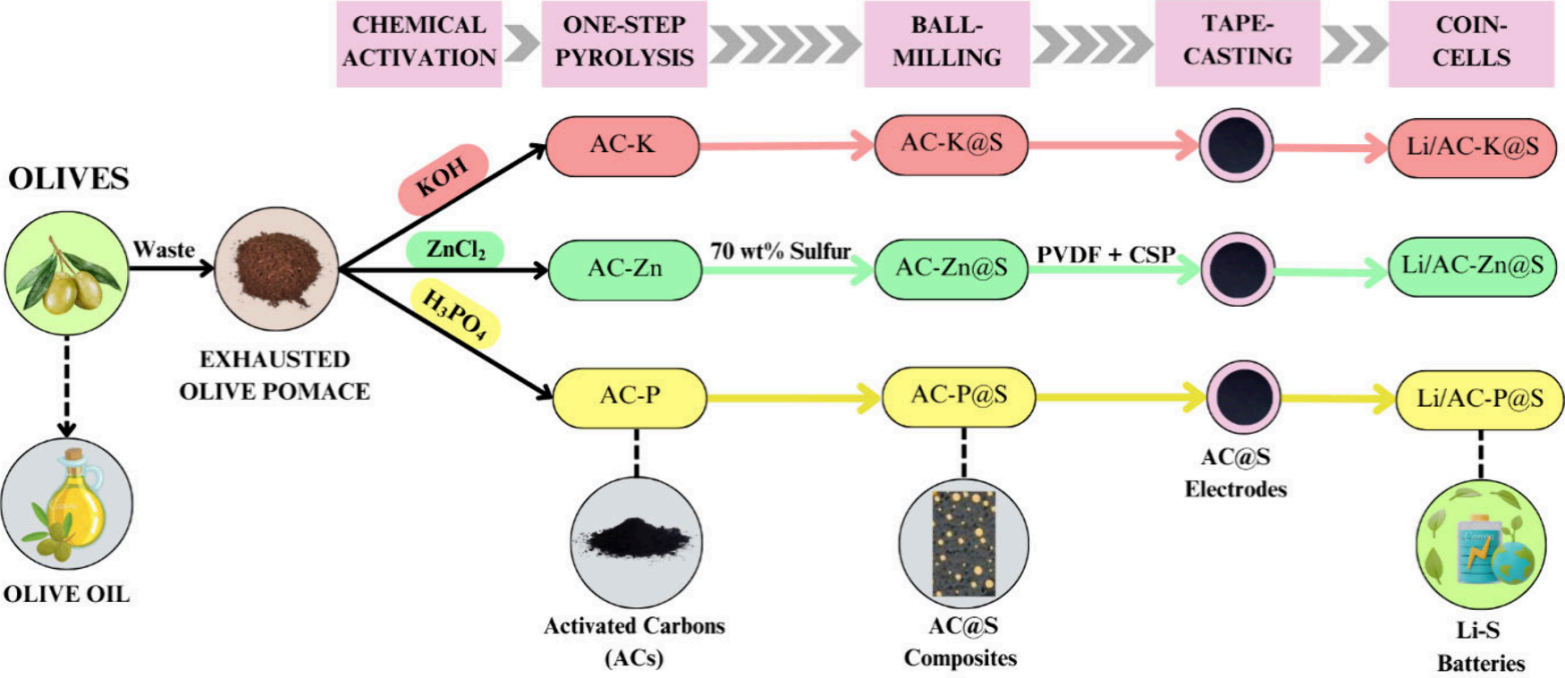
Scheme for the preparation of Li–S batteries using the *alpeorujo*-derived ACs.

## 3 Results and Discussion

### 3.1 Characterization of the AC Samples

A preliminary characterization of *alpeorujo* was carried out to assess its suitability of this potential as a raw material to produce highly porous ACs.^27^ Table S1 summarizes its main physicochemical properties. The *alpeorujo* exhibited a high moisture content (70%), making a predrying step necessary prior to any thermochemical treatment. In addition, it presented a slightly acidic pH, which may limit its direct application in biological valorisation processes such as composting or anaerobic digestion. In addition, the analysis revealed a notable diversity of elements in the *alpeorujo*. Particularly, it contains a significant amount of carbon, nitrogen, and phosphorus. These elements are of interest for both thermochemical and electrochemical applications. During activation process, these heteroatoms can be incorporated into the carbon matrix during activation, modifying the surface chemistry and, more importantly, improving the electrochemical properties of the resulting material. This is particularly relevant for applications in energy storage systems, specifically as a carbon host in Li–S batteries.^20,21^ Additionally, the volatile solids (VS) content reached 93%, indicating a high proportion of organic matter and reinforcing the suitability of *alpeorujo* for thermochemical valorisation. In contrast, the fixed solids (FS), associated with mineral content, were relatively low (∼7%), favoring the formation of porous carbon structures during activation process. Overall, these characteristics highlight *alpeorujo* as a promising precursor for the sustainable production of ACs, particularly for use as carbon host matrices in Li–S batteries.

The textural properties of the ACs were evaluated through nitrogen adsorption/desorption isotherm measurements (Figure 2a). According to the Brunauer–Deming–Deming–Teller (BDDT) classification, the isotherms for all synthesized materials exhibited type I and type IV profiles. This combined behavior corresponds to the typical curve of micro- and mesoporous solids.^22,23^ This dual pore system has been reported as highly desirable for its application as an efficient sulfur host in Li–S batteries, as it promotes both electrolyte impregnation and the mobility of Li^+^ ions between the electrodes.^24^ In addition, the bimodal microporous and mesoporous nature was confirmed by the pore size distribution graphs shown in Figure 2b. The results reveal that the main adsorption sites were in the range of micropores (Ø ≤ 2 nm) and small mesopores (around 10 nm). Notably, the AC-K sample exhibited the highest contribution of micropores compared to the other two AC samples. Similarly, the AC-P carbon showed a more balanced distribution between micro- and mesopores. In contrast, the AC-Zn sample scarcely exhibited mesoporosity, confirming the trend of zinc chloride as an AA to produce highly microporous ACs.^25^

**Figure 2:**
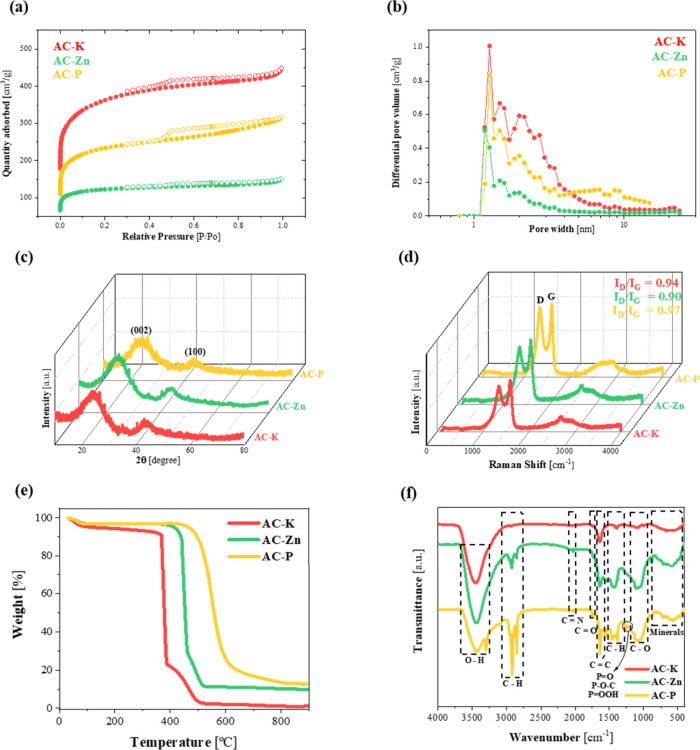
(a) N_2_ adsorption/desorption isotherm, (b) pore size distribution, (c) XRD patterns, (d) Raman spectrum, (e) TGA curves recorded under oxygen flow, and (f) FTIR spectrum of AC-K, AC-Zn, and AC-P samples.

Textural parameters were determined from the previous isotherms and summarized in Table S2. The AC-K sample exhibited the highest values for specific surface area and total pore volume (1830 m^2^/g and 1.05 cm^3^/g, respectively), indicative of a greater abundance of micropores. The AC-P sample also demonstrated high values for both *S*_BET_ and V_T_ (1058 m^2^/g and 0.68 cm^3^/g, respectively). In contrast, the AC-Zn sample showed considerably lower values for these parameters compared to the others. The trend of specific surface area values based on AA used was in line with previous studies.^26^ Each AA reacts differently with cellulose, hemicellulose, lignin, or polysaccharides in the carbon precursor, leading to various activation mechanisms that result in ACs with different values of *S*_BET_.^27^ In the literature, the most widely used AA is KOH, as it allows the development of extremely high specific surface areas, as in the case of AC-K, and the formation of pores, mainly micropores.^17^ During activation, most of the KOH is converted into K_2_CO_3_:^27^

1 $$2KOH\to K_{2}O+H_{2}O$$

2 $$K_{2}O+CO_{2}\to K_{2}CO_{3}$$

3 $$4KOH+C\to K_{2}CO_{3}+K_{2}O+2H_{2}$$

4 $$K_{2}O+C\to 2K+CO$$

5 $$K_{2}CO_{3}+2C\to 2K+3CO$$

The vapor of metallic potassium, generated by the reduction of K_2_CO_3_ or K_2_O with the carbon from the raw material, promoted the increase in total pore volume. This vapor could penetrate the carbon lattice, expand the aromatic layers, distort their structure, and create new pores. Additionally, it altered the electronic distribution of carbon atoms, generated more reactive sites, improved surface wettability, and reduced surface tension. Therefore, the action of the metallic potassium vapor, which is essential for pore development, did not occur with acidic or neutral AA, explaining the higher specific surface areas of ACs produced with KOH.^28^ During activation with ZnCl_2_, it operates as a dehydrating agent, reacting with the water generated from the scission of glycosidic bonds, leading to the elimination of hydroxyl and carbonyl groups.^29^ HCl is emitted as a gas, while the intermediate Zn_2_OCl_2_ decomposes into ZnCl_2_, ZnO, and H_2_O at temperatures above 500 °C. ZnCl_2_ and H_2_O are in the gaseous phase, and ZnO is formed as a byproduct.^30^

6 $$2ZnCl_{2}+3H_{2}O\to Zn_{2}OCl_{2}\cdot 2H_{2}O+2HCl↑$$

7 $$Zn_{2}OCl_{2}\cdot 2H_{2}O\to ZnCl_{2}↑+ZnO+2H_{2}O↑$$

The activation mechanism involving H_3_PO_4_ is described below (eqs 8–12).^31^ When H_3_PO_4_ undergoes dehydration, it releases water and generates phosphorus pentoxide (P_4_O_10_). This powerful oxidizing agent interacts with carbon, promoting the development of a complex system of micropores and mesopores, which contributes to a significantly increased specific surface area.^32^ In turn, these reactions promote the formation of elemental phosphorus, which can be incorporated into the carbon matrix as a heteroatom. This doping effect can enhance the material’s performance in various applications, such as energy storage.

8 $$2H_{3}PO_{4}\to H_{4}P_{2}O_{7}+H_{2}O$$

9 $$3H_{3}PO_{4}\to H_{5}P_{3}O_{10}+2H_{2}O$$

10 $$nH_{3}PO_{4}\to H_{n+2}P_{n}O_{3n+1}+\left(n-1\right)H_{2}O$$

11 $$H_{n+2}P_{n}O_{3n+1}\to P_{4}O_{10}+H_{2}O$$

12 $$P_{4}O_{10}+10C\to P_{4}+10CO$$

Nevertheless, in this study, all three samples showed a high contribution of microporosity, as observed in Figure 2b and confirmed by numerical values in Table S2.

In order to determine the structure of the ACs, XRD and Raman spectroscopy measurements were analyzed. Figure 2c illustrates that all the diffractograms exhibited two main peaks at 26° and 43° (2θ), which are characteristic of carbon materials with a graphitic structure. These broad bands correspond to the (002) and (100) crystallographic planes of graphite (PDF# 12-0212). This observation suggests that the obtained ACs had a low crystallinity structure, indicative of a relatively disordered graphitic arrangement. This quasi-amorphous structure is characteristic of ACs derived from *alpeorujo* ^33^ and other biomasses.^34^

Additionally, to determine the degree of disorder within the materials, Raman spectroscopy was conducted. The Raman spectra shown in Figure 2d revealed two main signals: the D band (∼1330 cm^–1^) and the G band (∼1595 cm^–1^), associated with structural defects and graphitic order, respectively.^35^ The intensity ratio between these bands (I_D_/I_G_) provided insight into the degree of disorder in the materials. In this case, this ratio was 0.94, 0.90, and 0.97 for AC-K, AC-Zn, and AC-P samples, respectively. These values, which are close to 1, signify a high degree of disorder and higher number of structural defects present in all the synthesized materials. The disorder in the structure of ACs was generated during the activation process, which involved both the removal of impurities and the development of pores. These processes transform the ordered arrangement of carbon atoms, creating a more random and disordered lattice. This structural alteration increases the presence of defects and disorder, which is reflected in an I_D_/I_G_ intensity ratio close to 1. Previous studies on biomass-derived ACs have shown similar results, confirming that activation, in addition to increasing the material’s porosity, also contributes to the formation of an amorphous structure.^36^

To quantify the fixed carbon content in the ACs, thermogravimetric measurements up to 900 °C were conducted in an oxidizing atmosphere, as shown in Figure 2e. The initial weight loss below 100 °C quantifies the moisture content present in the samples, with values detected below 5% in all cases. The main stage of weight loss was observed between 350 – 600 °C, associated with the combustion of carbon, the major component of the ACs. As shown in the thermogram, the sample activated with KOH exhibited a loss of carbon at a lower temperature. Many researchers attribute this to KOH lowering the activation reaction temperature, accelerating the removal of noncarbon elements, and increasing the pyrolysis reaction rate.^27^ The results showed that the three samples contained a high percentage of carbon, specifically, 93.2%, 86.9%, and 84.6% for AC-K, AC-Zn, and AC-P samples, respectively. Above 800 °C, the weight of all samples stabilized, reflecting the lower remaining mineral content for the AC-K sample. The higher carbon content in the AC-K sample reflects a greater chemical transformation of *alpeorujo* into carbon, which is consistent with the calculated production yields (Figure S1). The lower yield observed when KOH is used as an AA was a previously reported phenomenon,^37^ suggesting a deeper chemical action on the components of the *alpeorujo* for hydrolyzing, dehydrating, and thus attacking the biomass skeleton and creating pores. On the other hand, the activation strategy with H_3_PO_4_ provided the highest AC production yield, making it a promising option for potential industrial-scale implementation of the valorisation process. The higher yield achieved with H_3_PO_4_ activation is consistent with other studies, where the production yield sequence is like that observed in this work for *alpeorujo*.^26,38^ Additionally, as shown in Figure S1, the yield of generated AC was also calculated in terms of grams of AC per liter of wet alpeorujo, using Equation S2. Consistent with the dry-basis yield results, AC-P showed the highest production yield (86.5 g_AC_/L_*alpeorujo*_), followed closely by AC-Zn (79.1 g_AC_/L_*alpeorujo*_). In contrast, AC-K exhibited a significantly lowest yield (31.8 g_AC_/L_*alpeorujo*_), indicating a much less efficient conversion. As previously discussed, the low production yield associated with AC-K was mainly attributed to secondary reactions between KOH and specific components of the alpeorujo matrix, which led to considerable mass loss during activation. Furthermore, the excessive development of porosity in AC-K likely promoted to a decrease in material density, consequently limiting the amount of carbon that could be recovered per unit volume of raw material.

Table 1 shows the main elements present in each AC, as determined by XRF, except for nitrogen content, which was quantified from elemental analysis. There was a low presence of silicon (Si) in all three ACs, with AC-P exhibiting the highest concentration (1.18%) and AC-K the lowest (0.881%). As expected, AC-P, AC-Zn, and AC-K samples exhibited significantly higher proportions of phosphorus (P), chlorine (Cl), and potassium (K), respectively, due to the specific chemical AA used in each synthesis. Carbon was the predominant element, with AC-K showing the highest proportion (94.93%), followed by AC-Zn and AC-P, in accordance with the TGA results. Regarding nitrogen (N) content determined by elemental analysis, both AC-K and AC-P exhibited similar percentages, while the AC-Zn sample stood out with a nitrogen content of 1%. This result verifies the possibility of obtaining N-doped ACs derived from *alpeorujo*. The XPS analysis (Figure S2) of the ACs corroborates the detection of N as a dopant element in the carbon matrix, with signals corresponding to graphitic-N (401.7 eV), pyrrolic-N (400.0 eV), and pyridinic-N (398.6 eV).^39^ In the case of AC-P, additional peaks associated with P–O and P–C species were also observed, confirming the successful incorporation of phosphorus into the carbon framework. The presence of N and P heteroatoms in the carbon matrix is highly beneficial for its application as a cathode in Li–S batteries. These dopants can create polar sites that strongly interact with polar polysulfide species. Such interactions effectively suppress the shuttle effect, while the modified charge distribution and increased surface polarity imparted by the dopants facilitate improved redox kinetics during cycling, ultimately enhancing the electrochemical performance of the cells.^40^

**Table 1:** Content of the Main Elements Present in the Three AC Samples

**Element (%)**	**AC-K**	**AC-Zn**	**AC-P**
**Si**	0.88	1.01	1.18
**P**	–	–	4.10
**Cl**	0.59	4.79	0.02
**K**	1.25	0.74	0.63
**C**	94.93	88.92	83.01
**N**	0.64	1.02	0.61

Moreover, for identifying surface functional groups, FTIR spectroscopy was performed on the three ACs, as shown in Figure 2f. The different bands were designated based on previous studies.^41^ The wide band around 3300–3550 cm^–1^ presented in the three ACs corresponds to the O–H stretching vibrations. The low, medium, and high intensity bands for AC-K, AC-P, and AC-Zn, respectively, between 2962 and 2853 cm^–1^, are associated with the axial stretching of the C–H bonds. The peak at 2075 cm^–1^, most clearly observed in sample AC-Zn, is related to the stretching vibrations of C=N. The presence of the low intensity peak for the three ACs at 1742 cm^–1^ corresponds to the C=O stretching vibrations; the peak around 1633 cm^–1^ is associated with the stretch of C=C bonds; vibrations at 1458–1363 cm^–1^ are attributed to the in-plane deformation vibrations of aliphatic and aromatic groups (C–H); the wide bands around 1000 cm^–1^ correspond to C–O stretching in acids, alcohols, phenols, ethers, and esters; and finally, the small peaks between 500–800 cm^–1^ are related to the existence of minerals. It is worth noting that for the sample activated with H_3_PO_4_, a peak around 1255 cm^–1^ was observed, which can be attributed to the stretching vibrations of the P=O bond and the O–C bond in the P–O–C structure, as well as the presence of P=OOH.^42^ These bands are characteristic of P-containing compounds, whose presence has been confirmed by XPS and EDS (Figure S2 and S3, respectively). The presence of this element enables the production of N, P-doped AC, which can significantly enhance electrochemical performance by improving polysulfide anchoring and conductivity, making it highly effective as a cathode material in Li–S batteries.^43^

### 3.2 Characterization of the Sulfur-Based Composites (AC@S)

To confirm the presence of sulfur in the matrix of the ACs, an analysis using SEM/EDS was performed (Figure 3). SEM images revealed variations in particle size among the samples. The AC-Zn@S sample exhibited the largest particles, followed by AC-P@S. In contrast, the AC-K@S sample had the smallest particle size, with a very low mineral matter content (2%), making it the most porous sample. The obtained SEM images were similar to those reported in previous studies on biomass-derived ACs, showing amorphous materials with a high density of pores.^44^ Regarding the EDS mapping, the images demonstrated a homogeneous distribution of S in all three composites without observing agglomeration of particles, indicating that the infiltration method used was effective.

**Figure 3:**
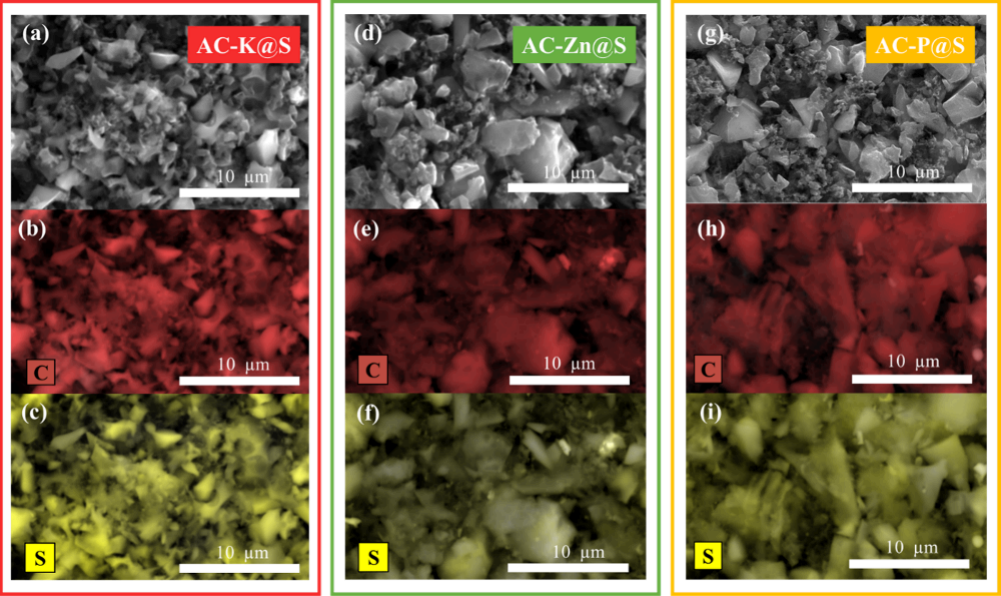
(a, d, g) SEM images and EDS elemental mapping of C (b, e, h) and S (c, f, i) for AC-K@S, AC-Zn@S, and AC-P@S composites, respectively.

In all the diffractograms of AC@S composites shown in Figure 4a, the characteristic crystallographic planes corresponding to pure orthorhombic sulfur, assigned to the *Fddd* space group (PDF# 42-1278), were clearly identified. The narrow and intense signals observed indicated that the samples exhibit a high degree of crystallinity, which is typical for materials primarily composed of sulfur.^45^ Furthermore, no peaks attributable to impurities were detected, suggesting that no secondary reactions occurred during sulfur deposition by wet ball milling, ensuring the purity of the final composites.

**Figure 4:**
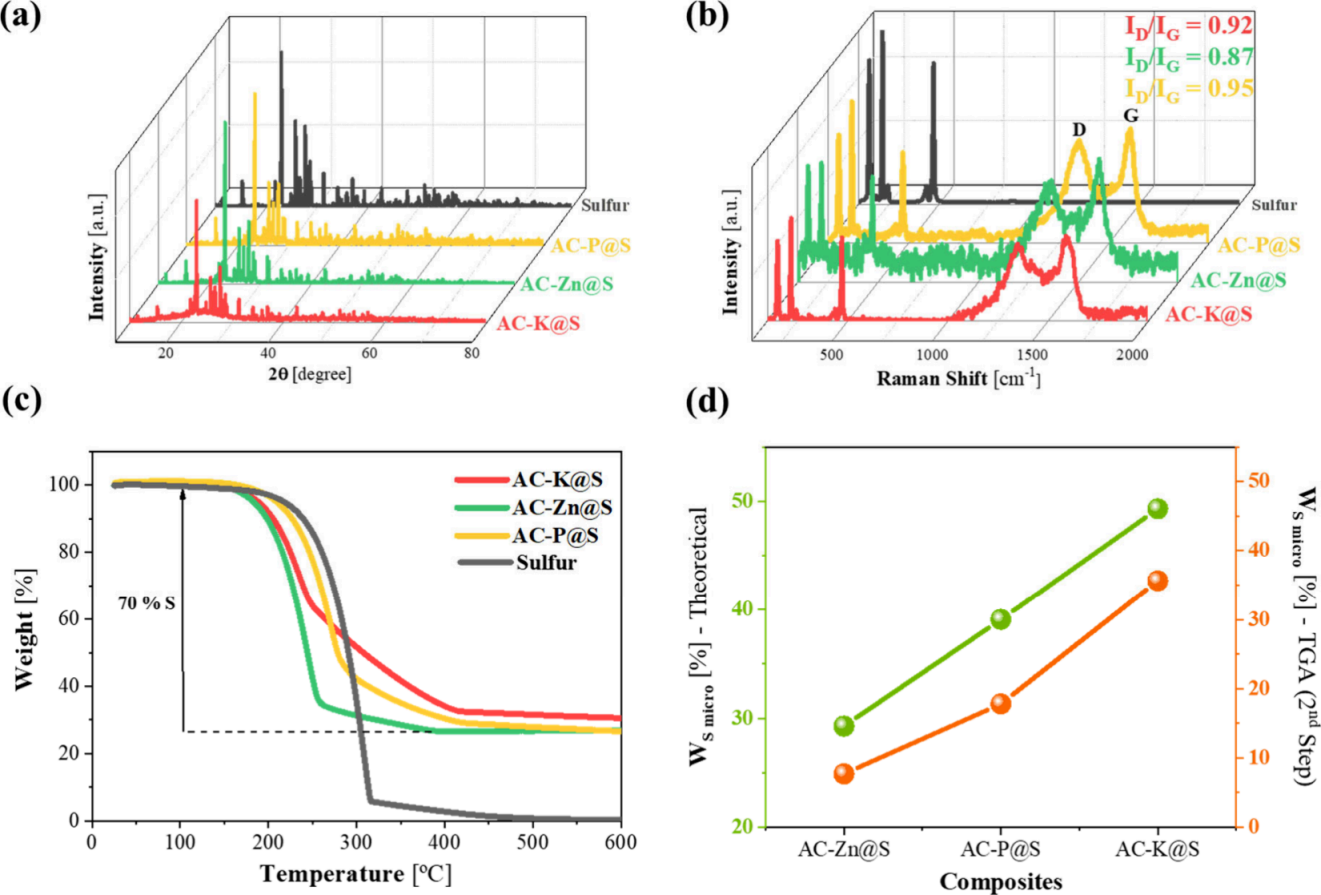
(a) XRD diffractograms, (b) Raman spectra, (c) TGA curves under N_2_ flow of the samples: AC-K@S, AC-Zn@S, and AC-P@S composites and pure sulfur, and (d) Correlation between the amount of S incorporated into the micropores, calculated theoretically and through TGA.

Continuing with the analysis of the structural properties of the composite samples, Raman spectra are shown in Figure 4b. The spectra once again revealed the D and G bands attributed to carbon materials, located around 1330 and 1595 cm^–1^, respectively, with no noticeable changes in the degree of structural ordering. However, additional signals emerged below 500 cm^–1^, specifically at 150, 219, and 474 cm^–1^, indicating the presence of S–S bonds corresponding to elemental sulfur.^46^

In order to accurately quantify the amount of sulfur fixed in the composites, thermogravimetric measurements were performed (Figure 4c) under an inert atmosphere for the different synthesized AC@S mixtures. A TGA of pure sulfur was also performed, confirming complete sulfur volatilisation by 350 °C. The AC@S composites exhibited an initial, negligible weight loss, corresponding to moisture, followed by the main weight loss occurring between 150 and 450 °C. This loss is attributed to the evaporation of sulfur present in the mixtures consistently around 70%, aligning with the proportion used during composite preparation. In all three cases, two stages of sulfur volatilisation were observed, which are associated with sulfur confinement in different porosities.^47^ Sulfur that is lost rapidly at lower temperatures (first stage) is located outside the pores or incorporated into larger pores. In contrast, sulfur loss occurring at higher temperatures (second stage) indicates sulfur trapped inside the pores or within carbon micropores, requiring more energy for volatilisation. This illustrates why the AC-Zn@S sample loses most of its sulfur during the first stage, as it has a low micropore volume (0.20 cm^3^/g). In contrast, for the other two samples (AC-K@S and AC-P@S), sulfur loss occurs at higher temperatures because a higher amount of sulfur is trapped in the micropores, due to their larger micropore volumes (0.47 and 0.31 cm^3^/g, respectively). Likewise, in Figure 4d, the direct relationship between the theoretical sulfur content incorporated into the micropores and the sulfur volatilized during the second stage of the TGA is represented. For this calculation, the following equation was applied as a theoretical S content that can be accommodated in the pores of the carbon matrix. These values were compared with the S content determined by TGA, as presented in Table S3.

13 $$W_{S}\;\left(\%\right)=\frac{\rho_{S}\cdot V_{T}}{\rho_{S}\cdot \left(V_{micro}+1\right)}\cdot 100$$

where *ρ*_*S*_ is the theoretical density of sulfur (2.07 g/cm^3^), *V*_*T*_ is the total pore volume, and *V*_*micro*_ is the micropore volume of the carbonaceous matrix.

### 3.3 Economic and Energy Analysis of the Process

The energy and economic balances performed to produce ACs from alpeorujo using different activating agents revealed significant differences in both energy demand and production costs. In addition, the production costs of the AC@S composites, obtained by mixing the ACs with sulfur (70 wt % sulfur), were also evaluated at the laboratory scale. The detailed costs, hypotheses, and results of these balances are summarized in Table S4.

In terms of energy consumption, the drying stage represented one of the main contributors, with a requirement of 1829 MJ/t of wet alpeorujo. However, the highest energy demand was observed during the pyrolysis stage, reaching 7942 MJ/t of wet alpeorujo (26473 MJ/t of dry alpeorujo), which constituted the dominant energy component of the process. In contrast, the grinding steps for both the raw materials and the AC@S composites required comparatively lower energy inputs, 400 MJ/t and 1296 MJ/t, respectively, relative to both dry materials. These findings indicate that pyrolysis is the primary target for optimization in terms of energy efficiency.

From an economic perspective, the analysis highlighted marked differences among the AA. AC-K exhibited the highest estimated production cost (€ 6.72/kg), mainly due to its low carbon yield, which increased the cost per unit of product despite the relatively low price of KOH (€ 790/t). In comparison, the use of ZnCl_2_ reduced the production cost to € 2.98/kg, while H_3_PO_4_ resulted in the lowest production cost (€ 1.98/kg), making it the most economically favorable option. When sulfur (70 wt %) was incorporated to produce AC@S composites, the cost per kilogram decreased substantially: € 2.41/kg for AC-K@S, € 1.29/kg for AC-Zn@S, and € 0.99/kg for AC-P@S. This reduction was attributed to the low cost of sulfur (€ 500/t) compared to activating agents and its high percentage in the composite, further strengthening the economic competitiveness of AC-P@S.

Overall, these results demonstrate that the economic feasibility of the process is strongly influenced by the choice of AA under equal production conditions. Therefore, optimization strategies should focus on reducing energy consumption during pyrolysis without compromising the final properties of the battery electrode base material. Moreover, the production cost of these materials is more competitive than that reported in previous studies,^48^ further reinforcing the potential of this approach for scalable and cost-effective electrode material manufacturing.

### 3.4 Electrochemical Measurements in Li–S Cells

Figure 5a, b, and c show the CV of Li/AC@S cells. For the three materials tested (Li/AC-K@S, Li/AC-Zn@S, and Li/AC-P@S), the reduction profile corresponding to the discharge process exhibited two distinct cathodic peaks. The first peak was observed at 2.4 V, which is attributed to the cleavage of the S_8_ sulfur ring and the subsequent formation of long-chain lithium polysulfides (eq 14). In contrast, the second peak, located at approximately 2.05 V, is associated with the transformation of long-chain lithium polysulfides into lithium sulfide (eq 15).^49^

14 $$2Li+nS↔Li_{2}S_{n},\left(n>4\right)$$

15 $$2\left(n-1\right)Li+Li_{2}S_{n}↔nLi_{2}S$$

**Figure 5:**
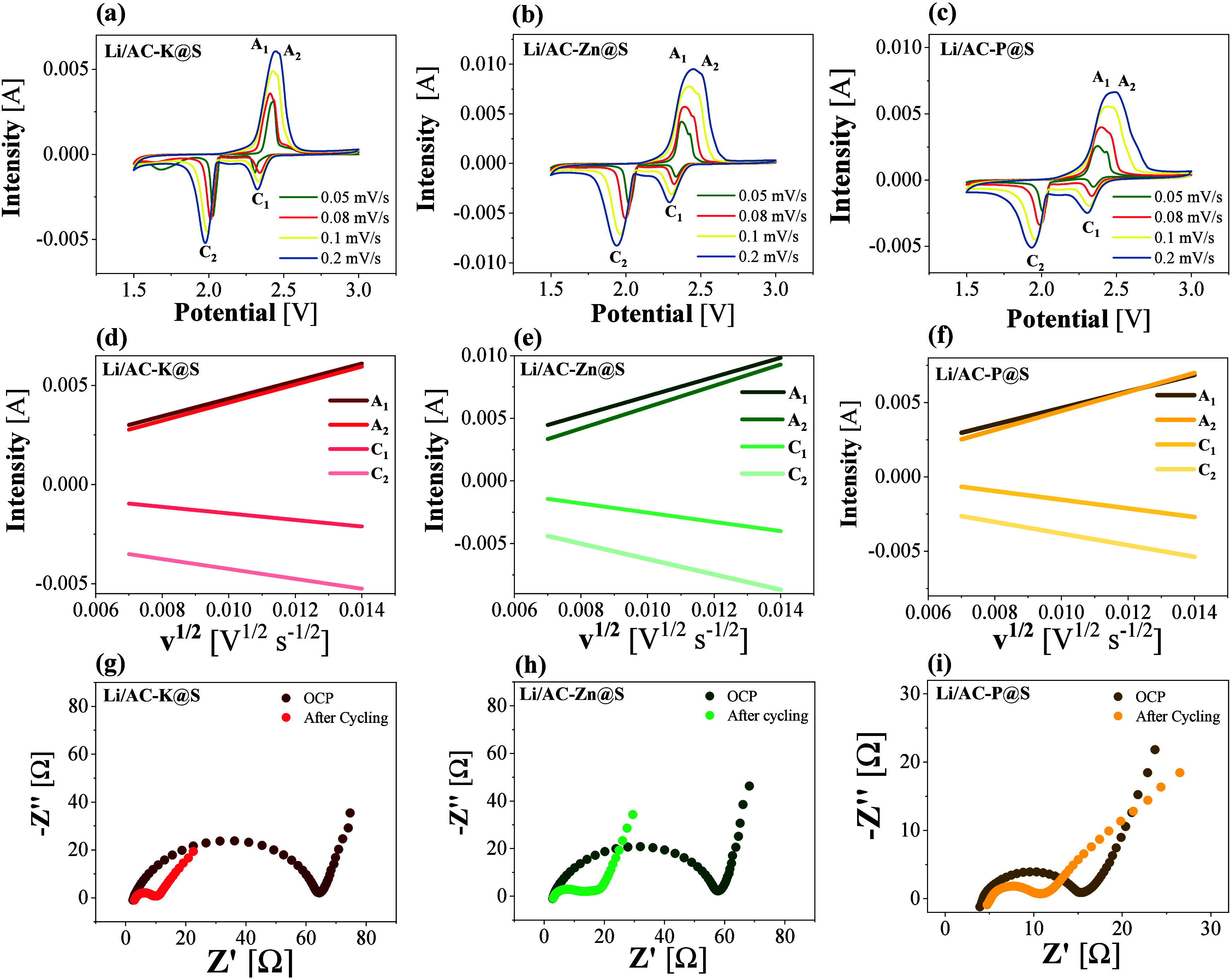
(a, b, c) Cyclic voltammograms at 0.05, 0.08, 0.1, and 0.2 mV/s, (d, e, f) graphical representation of linear fits of peak intensity (I_p_) vs the square root of the scan rate (v^1/2^) necessary for the calculation of Randles–Sevcik equation, and (g, h, i) Nyquist plots recorded by EIS at OCP and after 4 cycles of CV at 0.2 mV/s for Li/AC-K@S, Li/AC-Zn@S, and Li/AC-P@S cells.

During the charging process, at low scan rates (0.05 and 0.08 mV/s), two distinct but overlapping anodic signals could be clearly observed in the three cells studied, with peak voltages occurring around 2.25 and 2.35 V. These signals highlight that both above-mentioned oxidation reactions occur during charging, confirming the reversibility of the electrochemical process.

The diffusion coefficients of Li ions were calculated in each of the peaks described in the CVs using the Randles–Sevcik method, which states that the fitting of the maximum intensity of each signal is proportional to the square root of the velocity (eq 16).

16 $$I_{p}=2.69\cdot 10^{5}\cdot n^{3/2}\cdot A\cdot D_{Li}^{0.5}\cdot C_{Li}\cdot v^{0.5}$$

where *I*_*p*_ is the maximum intensity of each peak (A); *n* is the number of electrons involved in each process; *A* is the electrode area (cm^2^); *D*_*Li*_ is the diffusion coefficient of Li ions (cm^2^/s); *C*_*Li*_ is the concentration of Li ions in the electrolyte (M); and *v* is the scan rate (V/s) applied in each voltammetry experiment. Randles-Sevcik plots are shown in Figure 5d, e, and f, while Table S5 lists the Li^+^ diffusion values of the studied electrodes, which were calculated for each signal at different scan-rates. In all three cases, the diffusion coefficients were on the order of 10^–5^ and 10^–7^, with higher values compared to previously reported studies about electrodes derived from biomass tested in Li–S batteries.^50,51^ The high values of the Li diffusion coefficient indicate significant mobility of the ions within the electrode material. This high mobility correlates with the electrical conductivity measured for the cathodes by the four-point probe method (Table S6), suggesting that the porous structure of the material not only facilitated ionic transport but also provided an effective conductive network for the flow of electrons, primarily in AC-P@S and AC-K@S composites.^52^ Both the Randles-Sevcik analysis in CV and the direct conductivity measurements by four-point probe on the electrodes confirmed a superior ionic and electronic diffusion capacity of the composite based on AC-P. This confirms that the presence of P as a heteroatom dopant in the N, S-doped carbon structure can enhance conductivity in the electrode material.

The EIS measurements recorded in Figure 5g, h, and i were analyzed to gain further insights into the electrochemical behavior of the prepared cells. These graphs are represented using Nyquist diagrams, showing the behavior of the cells both at open-circuit potential (OCP) and after 4 cycles of CV. The intersection of the semicircular arc at high frequencies with the real Z’ axis reflects the electrolyte resistance, whereas at intermediate frequencies, the associated semicircle represents the charge transfer resistance (R_ct_). Upon comparing the R_ct_ of the various electrodes examined, it is evident that both Li/AC-K@S (R_ct_ = 62.3 Ω) and Li/AC-Zn@S (R_ct_ = 55.2 Ω) cells exhibited substantially higher resistance compared to the Li/AC-P@S (R_ct_ = 11.7 Ω) cell. Therefore, the EIS analysis confirms the improved conductivity properties for the AC-P carbon-based composite, in agreement with the Randles-Sevcik results and four-point conductivity measurements. Moreover, an analysis of the semicircles from the same samples (OCP and after CV) revealed a notable reduction in R_ct_ after several cycles of CV. The resulting values were 8.1 Ω for Li/AC-K@S, 15.8 Ω for Li/AC-Zn@S, and 6.1 Ω for Li/AC-P@S, indicating improved charge transfer efficiency with cycling. In Li–S battery technology, it is common to observe a sharp decrease in R_ct_ after CV measurements.^53^ This phenomenon is due to the activation of the cell, evidenced by the anodic and cathodic reactions during CV, as well as the complete saturation of the cathode with the electrolyte, which enhances the transfer of Li ions across the cell.

Figure 6 illustrates the galvanostatic charge and discharge (GCD) profiles for the prepared Li–S cells at 0.1C-rate. Both discharge and charge curves for all tested cells clearly exhibited two well-defined plateaus. During the discharge process, these plateaus occurred at approximately 2.38 and 2.10 V. The first plateau at 2.38 V is associated with the faster kinetics of the formation of long-chain polysulfides. The second plateau at 2.10 V, with slower kinetics, corresponds to the reduction of polysulfides to lithium sulfide. Regarding the charging process, the profiles also exhibited two plateaus at around 2.25 and 2.35 V. These plateaus are observed at closely related potentials, correlating with the overlap of anodic peaks observed during the CV data shown in Figure 5a, b, and c. The similarity between the charge plateaus and the anodic peaks suggests a direct correlation between the charge/discharge behavior and the electrochemical reactions occurring within the cell. Based on these profiles, the hysteresis in the fifth cycle was calculated considering the average voltage, with the Li/AC-P@S cell exhibiting the lowest polarization (0.17). Therefore, this cell is expected to offer superior efficiency and electrochemical performance. Comparatively, the hysteresis was calculated at different rates (Figure S4). It was observed that the hysteresis voltage increased with higher current densities, while the Li/AC-P@S cell consistently demonstrated the lowest hysteresis values in all cases.

**Figure 6:**
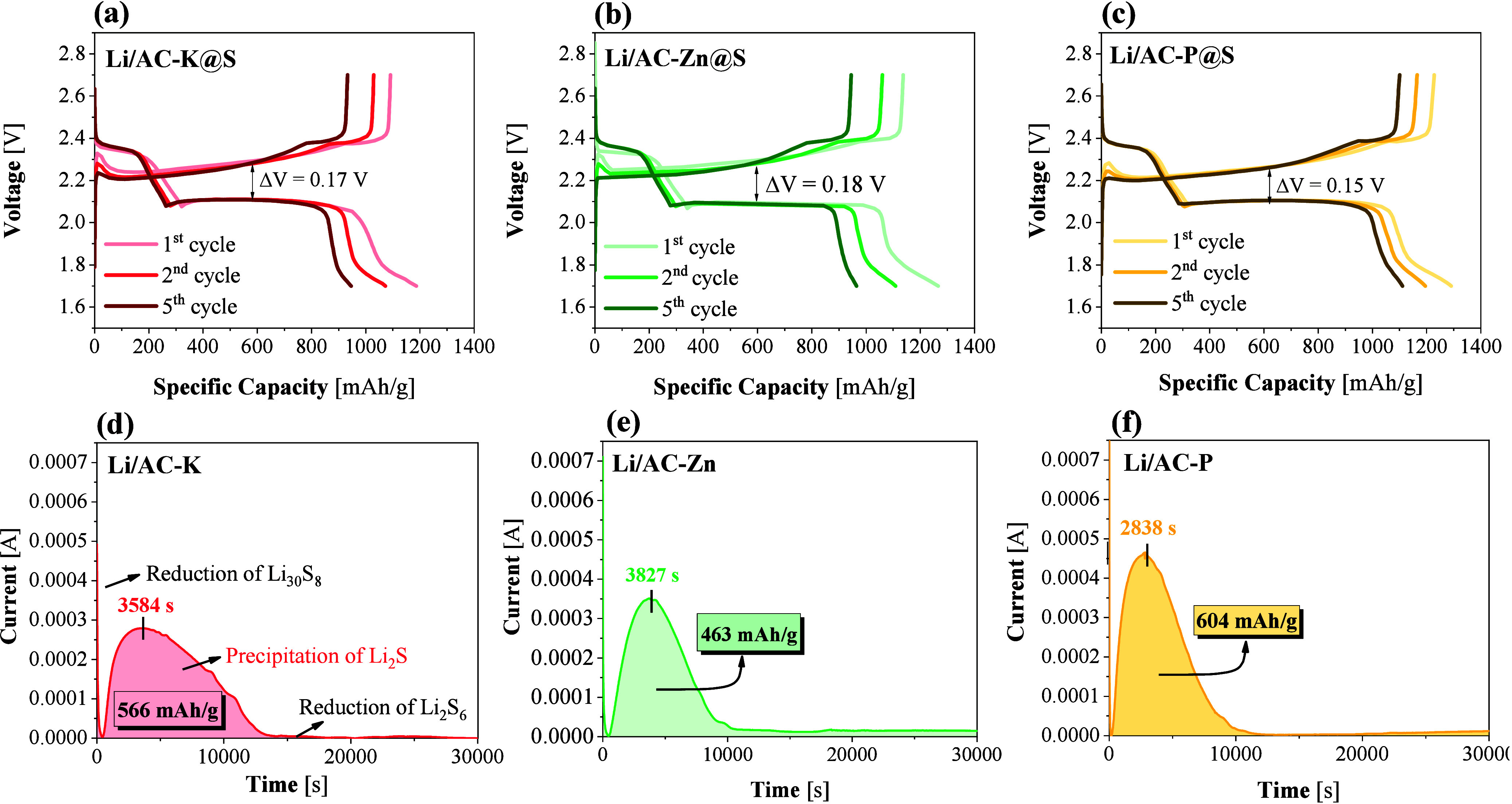
Charge/discharge profiles at 0.1C rate of the (a) Li/AC-K@S, (b) Li/AC-Zn@S, and (c) Li/AC-P@S cells. Potentiostatic discharge curves of Li_2_S precipitation at 2.01 V of (d) Li/AC-K, (e) Li/AC-Zn, and (f) Li/AC-P.

Potentiostatic discharge measurements were implemented to evaluate the nucleation and decomposition behavior of Li_2_S in the Li/AC@S cells, and results are shown in Figure 6d, e, and f. The AC-P-based electrode demonstrated the highest precipitation capacity of Li_2_S with a value of 604 mAh/g, exceeding that of the Li/AC-K electrode (566 mAh/g) and the Li/AC-Zn electrode (463 mAh/g). These results indicate superior catalytic activity of the Li/AC-P electrode in promoting Li_2_S nucleation. Furthermore, this higher catalytic activity is supported by the reduction of the time required for the initiation of the Li_2_S nucleation process.^54^ This result demonstrates the positive effect of N and P dual doping in the carbon matrix achieved through activation with H_3_PO_4_. In addition, Li_2_S dissolution measurements (Figure S5) further confirmed that the Li/AC-P electrode exhibited the highest capacity during the dissolution process (705 mAh/g), compared to Li/AC-K (665 mAh/g) and Li/AC-Zn (674 mAh/g), evidencing its enhanced reversibility and catalytic ability for the Li_2_S/Li_2_S_X_ conversion. This improved dissolution behavior was consistent with the positive effect of N and P dual doping, which generates polar sites and optimizes charge distribution in the carbon framework, thereby facilitating both polysulfide binding and accelerated redox kinetics.

To provide a comprehensive analysis of the specific capacities achieved by each AC-based electrode, the galvanostatic measurements are presented in Figure 7. This figure illustrates both the specific capacity and Coulombic efficiency of the Li–S cells as a function of cycle number. The cells were tested within a constant voltage window of 1.7 and 2.7 V across three different current densities: 0.2C (Figure 7a), 0.5C (Figure 7b), and 1C (Figure 7c). The first five charge and discharge cycles were conducted at lower current densities, specifically at 0.1C, with the aim of performing an activation stage for the cell. This initial low-current cycling helped to stabilize the cell’s performance by allowing for the formation of a solid electrolyte interphase (SEI) and by optimizing the electrochemical processes before subjecting the cell to higher current densities. In all cases, an initial capacity loss was observed; this phenomenon is known as the cell activation period.^55^ In Figure 7a, the cells cycled at 0.2C exhibited good stability over 150 cycles, with Coulombic efficiency values close to 100%. Among them, the Li/AC-P@S cell stood out with the highest specific capacity of around 1070 mAh/g, significantly outperforming the capacities of Li/AC-K@S and Li/AC-Zn@S, which were around 810 mAh/g. When cycled at 0.5C (Figure 7b), the cells also maintained good stability over 300 cycles and continued to exhibit Coulombic efficiency around 100%. At this higher C-rate, the three cells exhibited slightly lower capacity compared to those cycled at 0.2C. However, Li/AC-P@S still achieved the highest specific capacity, averaging 988 mAh/g. Finally, at a current density of 1C (Figure 7c), as expected, all cells showed even further lower specific capacity. The differences between cells became less pronounced, achieving values of 688, 653, and 592 mAh/g for Li/AC-P@S, Li/AC-K@S, and Li/AC-Zn@S cells, respectively. The overall electrochemical performance trends observed for the cells at different current densities were consistent, highlighting the superior performance of Li/AC-P@S cell under different conditions. The high performance was mainly due to the combined synergistic effect derived from the optimal textural properties of the AC treated with H_3_PO_4_ and the presence of P as a heteroatom electrocatalyst in the AC. Its high specific surface area (1058 m^2^/g) allowed for more uniform dispersion of sulfur, promoting greater interaction with the electrolyte, thus contributing to a higher charge and discharge capacity of the battery.^56^ Among the ACs studied in this work, it is true that AC-P does not exhibit the highest *S*_BET_; however, it offers an optimal micromesoporous structure for sulfur confinement, as has been suggested in another research paper.^57^ At the same time, previous studies have also shown that P-doped materials improve electrochemical performance of Li–S batteries.^58,59^

**Figure 7:**
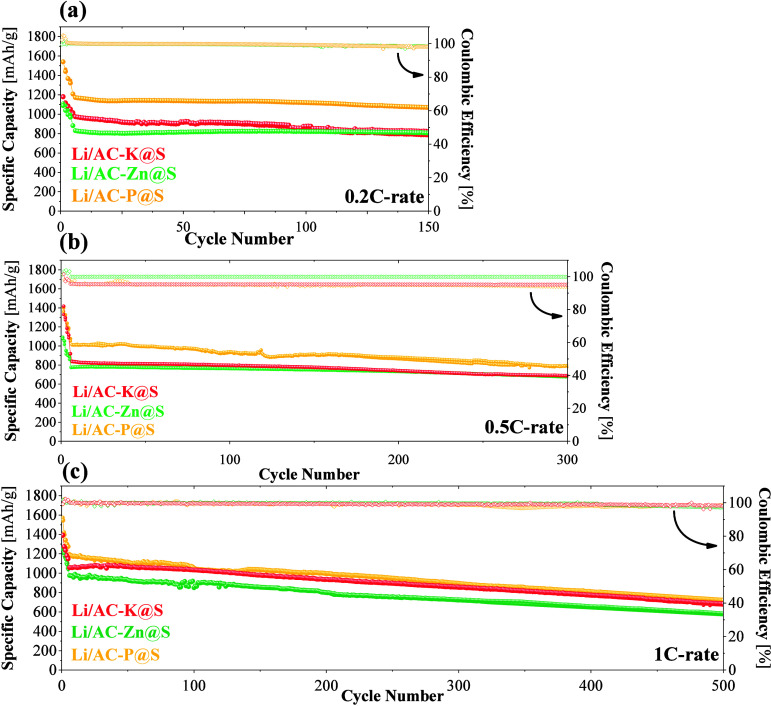
Long-term cycling of the different Li–S cells at (a) 0.2C, (b) 0.5C, and (c) 1C-rate.

Figure 8a presents the areal specific capacity over 150, 300, and 500 cycles at C-rates of 0.2, 0.5, and 1C for the three cells configurations, clearly showing that the Li/AC-P@S cell maintained superior specific capacity. The radar plot (Figure 8b) compares the specific capacity values at 0.2C, 0.5C, and 1C, the capacity decay rate, and the Coulombic efficiency at 1C after 100 cycles. This visualization enables a straightforward comparison of the electrochemical behavior among these three systems, highlighting the superior performance of the Li/AC-P@S cell, which achieved capacities in the range of 1000 – 1100 mAh/g across all tested rates. Regarding Coulombic efficiency, all three cells exhibited values close to 100%, demonstrating that the batteries operate optimally, with no significant capacity losses during the charge and discharge cycles.

**Figure 8:**
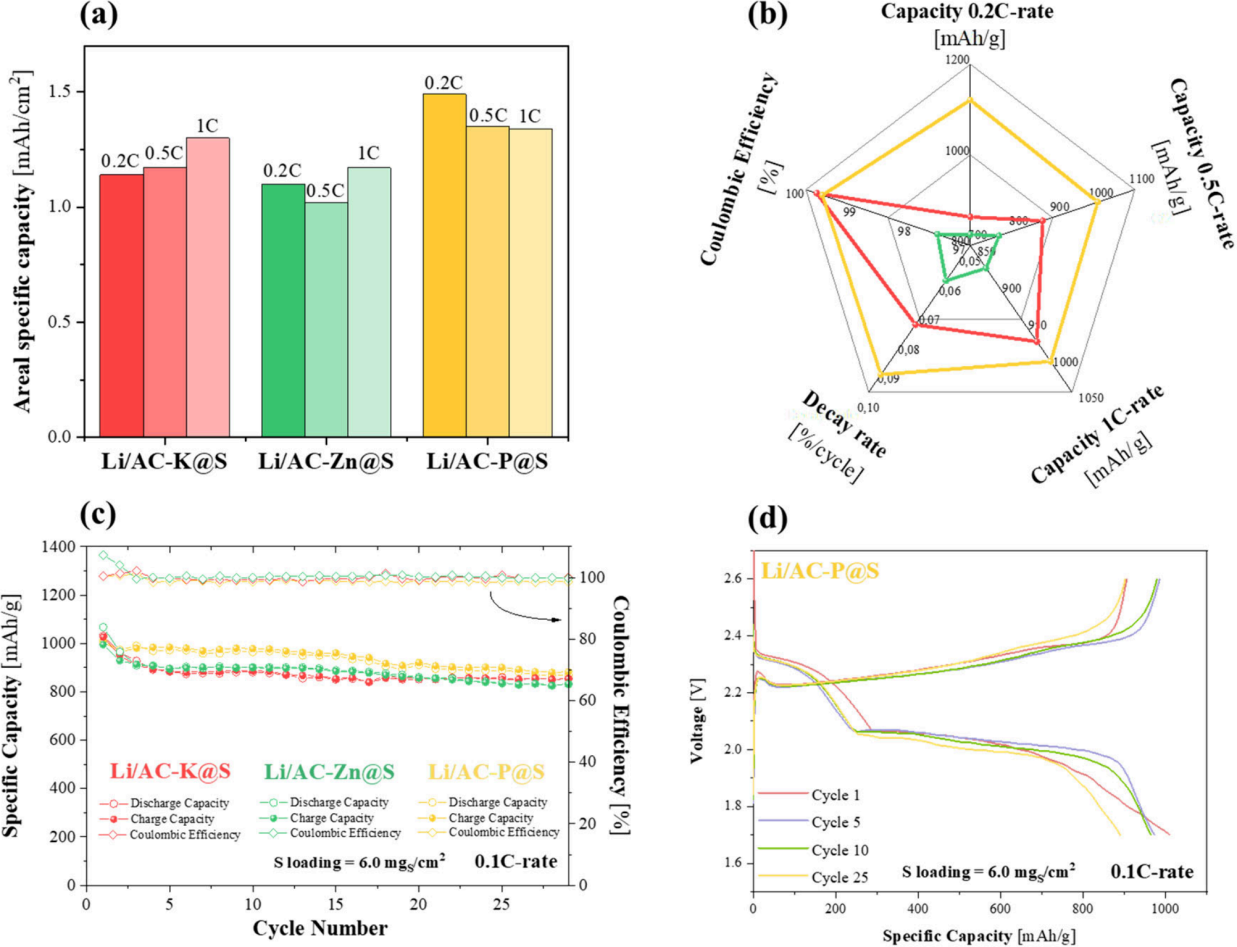
(a) Areal specific capacity over 150, 300, and 500 cycles at 0.2, 0.5 and 1C-rate, (b) Radar plot of electrochemical performances, (c) Cycling of the different Li–S cells at C/10 with high sulfur loading, and (d) Charge–discharge profiles of the Li/AC-P@S electrochemical cell at 0.1C with a S-loading of 6.0 mg_S_/cm^2^.

To evaluate the practical feasibility of sulfur-based cathodes in lithium–sulfur batteries, additional electrochemical tests were conducted under conditions that simultaneously combined high sulfur loading and low electrolyte content, as shown in Figure 8c. Such stringent conditions are critical to approaching the gravimetric and volumetric energy densities required for real-world applications. Specifically, Li/AC-K@S, Li/AC-Zn@S, and Li/AC-P@S cells were evaluated at high sulfur loading (6.0 mg_S_/cm^2^) with an electrolyte-to-sulfur ratio (E/S) of only 11 μL/mg_S_. All three systems exhibited relatively stable cycling behavior with minimal capacity fading (0.56%/cycle for Li/AC-K@S, 0.55%/cycle for Li/AC-Zn@S, and 0.48%/cycle for Li/AC-P@S), even under these demanding parameters. Notably, as anticipated, the Li/AC-P@S cell demonstrated superior performance, maintaining a high specific capacity (above 1000 mAh/g), excellent capacity retention over cycling (2.97%/cycle), and near-100% Coulombic efficiency. These results highlight the strong potential of the AC-P@S configuration for practical Li–S battery applications operating under high S-loading and lean-electrolyte regimes. Moreover, the close overlap between charge and discharge capacities throughout cycling (Figure 8d) confirms the high reversibility of the electrochemical process and efficient utilization of sulfur under these realistic operating conditions. The corresponding charge–discharge profiles of the Li/AC-K@S and Li/AC-Zn@S cells are shown in Figure S6.

To further justify the superior electrochemical performance of certain electrodes and to assess the effective polysulfide retention in the ACs, polysulfide adsorption measurements were conducted inside the glovebox using a 0.5 mM Li_2_S_6_ solution (reference sample). Continuous monitoring of the absorbance at the maximum wavelength of 420 nm enabled the evaluation of polysulfide adsorption capacity. As depicted in Figure 9, the absorbance of the reference Li_2_S_6_ solution remained constant over time, as anticipated. In contrast, the measurements involving the Li_2_S_6_ solution in contact with the ACs demonstrated a noticeable decrease in absorbance, indicating significant polysulfide adsorption by the ACs. The results clearly demonstrate that the AC-P sample adsorbs polysulfides more effectively and at an earlier stage compared to AC-K and AC-Zn samples. This enhanced LiPS adsorption correlates with improved electrochemical performance, as it reduces the shuttle effect—one of the main causes of capacity fade.^60^ Nonetheless, additional factors such as interphase evolution (SEI formation), parasitic side reactions, and structural degradation of the porous host may also contribute to long-term capacity decay and should be considered when evaluating the observed improvements.^61^ In contrast, the AC-Zn sample exhibited a notably lower polysulfide adsorption capacity. Consequently, these findings align with the electrochemical behavior observed in the cells presented in Figure 7, where in all cases the cells incorporating AC-P showed superior electrochemical performance compared to those with AC-K and AC-Zn. Figure 9b shows the visual appearance of the Li_2_S_6_ solutions after 120 min of interaction with the AC-K, AC-Zn, and AC-P samples, highlighting the different degrees of decolorization as an indication of LiPS adsorption. To gain deeper insight into the adsorption dynamics, Figure 9c–e displays the time-resolved UV–vis spectra of the Li_2_S_6_ solution in contact with each AC sample. A progressive decrease in the intensity of the characteristic absorption peaks centered around 420 nm is observed in all cases, confirming the gradual removal of Li_2_S_6_ species from the solution. Notably, the rate and extent of absorbance decay differ among the samples, with AC-P exhibiting the most pronounced and rapid reduction, followed by AC-K and then AC-Zn, in line with the trends observed in Figure 9a. These results further corroborate the superior adsorption capacity of AC-P for LiPSs.

**Figure 9:**
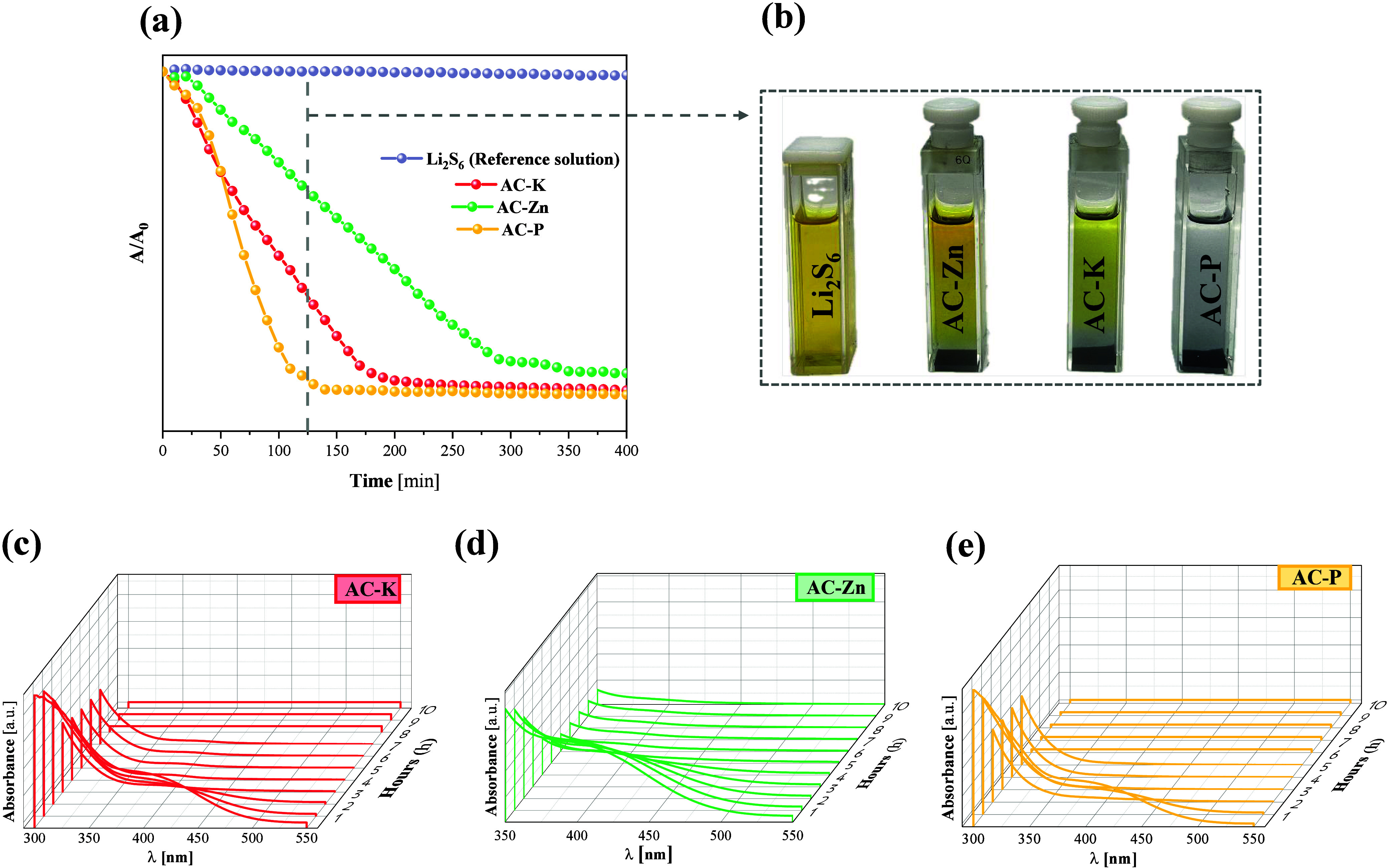
(a) Temporal evolution of the absorbance of Li_2_S_6_ solution in contact with the AC-K, AC-Zn, and AC-P samples, measured using UV–vis spectroscopy at 420 nm; (b) Image of Li_2_S_6_ solutions at 120 min; and dynamic evolution of UV–vis spectra for Li_2_S_6_ solution with (c) AC-K, (d) AC-Zn, and (e) AC-P.

Complementary to the LiPS adsorption test, XPS analysis on the AC samples after the adsorption process was performed. This analysis enabled a detailed investigation of how AC-materials interact with the polysulfides present in the solution. Figure S7 shows the spectra obtained for the S *2p* region of AC-K, AC-Zn, and AC-P samples, where the two typical signals of this element, deconvoluted into five components, were observed. These signals were assigned to specific chemical species, such as S–H, S–C–S, C=S, and the complex polysulfides C–SO_2_–C and C–SO_3_–C.^62^ The presence of these signals confirms not only the effective adsorption of the polysulfides on the electrode surface but also the chemical interaction between the functional groups of the electrode and the sulfide species. This behavior reconfirms that the electrode design favors a high affinity toward LiPSs, which is key to improving stability and performance in the electrochemical applications mentioned above.

Finally, Table 2 presents a comparison of the electrochemical performance of the Li/AC-P@S cell, which exhibited the best results in this study, with a range of carbon-based cathode hosts for Li–S batteries, including predominantly Acs derived from agricultural biomass. Several aspects clearly highlight the advantages of our approach in terms of performance, sustainability and scalability. First, from a synthesis standpoint, most reported biomass-derived ACs require multistep and time-consuming procedures, often involving high consumption of activating agents (biomass:AA ratios up to 1:4). In contrast, our process adopts a simpler and one-step calcination with a significantly lower AA requirement (biomass:AA ratio of 2:1), thus minimizing reagent use, waste generation, and environmental footprint, in accordance with green chemistry principles. Additionally, we have previously characterized the liquid effluents generated during the AC purification stage,^19^ showing that they are predominantly inorganic waters, mainly due to the presence of metal ions that are effectively removed from the *alpeorujo* during the process. This indicates that the process enhances the purity of the final carbon product and reduces its environmental impact. Second, in terms of electrochemical performance, despite having a lower sulfur content compared to some reported carbons (for example from pistachio shells or tea leaves), the Li/AC-P@S cell exhibited an outstanding specific capacity (1121 mAh/g) and the highest long-term stability among the carbon materials listed (688 mAh/g after 500 cycles). This corresponds to a decay rate of only 0.07% per cycle, the lowest of all compared cases, confirming highly efficient sulfur utilization and remarkable cycling stability under demanding conditions. Third, while some of the highest-performing Li–S cathodes reported in the literature rely on costly carbon matrices such as carbon nanotubes^63^ or graphene aerogels^64^ or on complex synthesis techniques with limited scalability, the AC-P@S electrodes presented in this work are produced through a simple, low-cost, and easily scalable process using agro-industrial waste as the raw material. This not only ensures economic viability for large-scale production but also strengthens the environmental credentials of the technology by valorizing a problematic waste stream and avoiding energy-intensive or chemically aggressive treatments.

**Table 2:** Electrochemical Performance of Different Carbon Materials for Li–S Batteries

Carbon material	Preparation method	Ratio of biomass: AA	Sulfur content (%)	Initial capacity (mAh/g)	Final capacity (mAh/g) (Cycle)	Rate (C)	Decay rate (%/cycle)	ref.
Rice husk derived AC	i) Precarbonization at 350 °C 2 h, (ii) manual mixing with ZnCl_2_, (iii) pyrolysis at 600 °C 3 h, and (iv) washed with water, 1 M HNO_3_, and 1 M HF	1:1.5	60	1057	518 (100)	0.2	0.51	^65^
Avocado shell derived AC	i) Precarbonization at 800 °C 2 h, (ii) refluxed in HNO_3_ at 100 °C	1:1	—	1300	530 (100)	0.1	0.53	^66^
Cherry pit derived AC	i) Mechanical activation with H_3_PO_4_, (ii) pyrolysis at 800 °C, (iii) washing stage	1.76	67	550	410 (200)	0.5	0.13	^32^
Pistachio shell derived AC	i) Impregnation with H_3_PO_4_ at 850 °C 3 h, (ii) pyrolysis at 800 °C 4 h, (iii) washed with water	1:1	80	1193	570 (300)	0.1	0.17	^67^
Corncob derived AC	i) Precarbonization at 400 °C 3 h, (ii) impregnation with KOH for 2 h, (iii) pyrolysis at 850 °C 3 h, (iv) washed with water	1:4	50	1252	664 (200)	0.3	0.23	^68^
Tea leaves derived AC	i) Washed with ethanol and HCl, (ii) precarbonization at 600 °C 2 h, (iii) impregnation with KOH, (iv) pyrolysis at 900 °C 2 h, and (v) washed with 1 M HCl, and water	1:3	71	1350	745 (100)	0.2	0.45	^69^
Pomelo peels derived AC	i) Precarbonization at 500 °C 5 h, (ii) impregnation with KOH for 8 h, (iii) pyrolysis at 600 °C for 2 h, and (iv) washed with water.	—	60.1	1258	750 (100)	0.2	0.4	^70^
Carbon nanotubes	Fluidized bed chemical vapor deposition (CVD) method	—	67	1249	800 (100)	0.1	0.64	^63^
Graphene aerogel	i) NiCo-LDH (20 mg) and NGS (50 mg) were dispersed in a 1:1 water/ethanol mixture, (ii) the dispersion was heated at 90 °C for 4 h in a Teflon-lined reactor, and (iii) the resulting mixture was freeze-dried for 24 h.	—	66	1190	1143 (100)	0.2	0.05	^64^
*Alpeorujo* derived AC	i) Manual mixing with H_3_PO_4_, (ii) pyrolysis at 900 °C 1 h, and (ii) purification with 6 M HCl	2:1	70	1121	688 (500)	1	0.07	This work

Overall, these combined factors—outstanding electrochemical performance, process simplicity, low cost, and minimal environmental footprint—position *alpeorujo*-derived ACs as a high-value, green, and industrially viable alternative for advanced energy storage applications in Li–S batteries.

## 4 Conclusions

This study demonstrates an efficient and sustainable strategy for upcycling *alpeorujo*, a highly polluting agro-industrial residue, into ACs via a straightforward, low-impact, and scalable process. Porous AC with high carbon content (around 80%) were obtained using minimal amounts of AA (biomass:AA = 2:1), in line with green chemistry principles by reducing reagent consumption, energy input, and waste generation. The inherent nitrogen content of *alpeorujo* allowed for self-doping of the carbon matrix, while activation with H_3_PO_4_ also introduced phosphorus, both enhancing surface functionality without requiring postsynthetic modifications. Additionally, among the synthesized materials, due to its cost and properties, H_3_PO_4_ emerges as a particularly promising candidate, offering a compelling balance between cost efficiency and overall process performance (€ 1.98/kg_AC‑P_ and € 0.99/kg_AC‑P@S_). These materials were subsequently combined with sulfur via wet milling, yielding composites with up to 70% sulfur loading for application as cathode materials in Li–S batteries. Including the systems tested, Li/AC-P@S delivered a remarkable initial capacity of 1121 mAh/g at 1C and exceptional cycling stability, retaining 688 mAh/g after 500 cycles, with near-perfect Coulombic efficiency and minimal capacity decay (0.07% per cycle). Under high sulfur loading cycling, the Li/AC-P@S system once again stands out for maintaining capacities close to 1000 mAh/g with excellent stability over the cycles. The superior behavior of AC-P was mainly due to two factors. First, its optimized pore distribution promotes electrolyte access and LiPSs retention. Second, phosphorus doping enhances electrical conductivity and promotes a more efficient nucleation process, contributing to greater electrochemical stability. These results exceeded current literature values, positioning this work as a promising, cost-effective, and sustainable approach. Overall, the proposed method exemplifies the integration of waste valorisation and sustainable energy storage, promoting circular economy models and advancing the development of environmentally responsible battery technologies.

## Supplementary Material



## Data Availability

The data supporting this study’s findings are provided within the article and its Supporting Information.

## Notes

Activated carbon yield data; XPS spectra for AC-K, AC-Zn, and AC-P samples; SEM images and EDS mapping of AC-P; hysteresis voltage from charge/discharge profiles; potentiostatic charge curves of Li_2_S dissolution for Li/AC-K, Li/AC-Zn, and Li/AC-P; charge–discharge profiles of the Li/AC-K@S and Li/AC-Zn@S electrochemical cell at 0.1C with S-loadings of 6.0 mg_S_/cm^2^; XPS spectra of S *2p* region after LiPS adsorption; physicochemical characterization of *alpeorujo*; textural properties of AC samples; trapped sulfur content by theoretical and TGA methods; economic and energy balances for ACs and AC@S composites; Li^+^ diffusion coefficients from Randles-Sevcik equation; electrical conductivity from four-point probe measurements. (PDF)
