# Supplementary material for: Effect of the Activation Agent on Carbons Derived from Exhausted Olive Pomace as Sulfur Hosts in Sustainable Lithium–Sulfur Batteries
Source: ACS Appl Mater Interfaces. 2025 Aug 25;17(35):49594–611. doi: 10.1021/acsami.5c12218 (PMC12424026; doi:10.1021/acsami.5c12218)
Supplement: Supplementary file 1 [file am5c12218_si_001.pdf]

Supporting information

Effect of the activation agent on carbons derived from exhausted olive pomace as sulfur hosts in sustainable lithium-sulfur batteries

Hansi Martínez-Alvarenga[a,b], Azahara Cardoso-Almoguera[a], Mª Carmen Gutiérrez[a,b], Almudena Benítez[a,*], Mª Angeles Martín[a,b], Alvaro Caballero[a]

[a] *Dpto. Química Inorgánica e Ingeniería Química, Instituto Químico para la Energía y el Medioambiente (IQUEMA), Universidad de Córdoba, 14071, Córdoba, Spain*

[b] *Campus de Excelencia Internacional Agroalimentario ceiA3, Universidad de Córdoba, Campus Universitario de Rabanales, N-IV, km 396, Córdoba, 14071, Spain*

*Corresponding author: A. Benítez (q62betoa@uco.es; Tel.: +34–957218467)

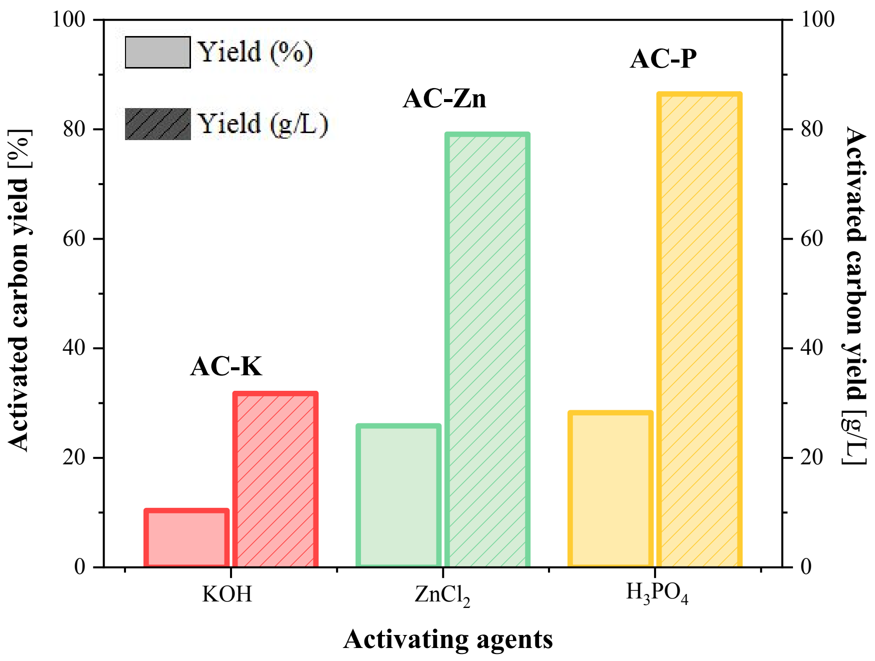

Figure S1. Activated carbon yields.

The experimental AC yields were calculated using the following equations:

$$Yield\ (Dry\ basis, \%) = \frac{W_C}{W_O} \cdot 100 \quad (Eq. S1)$$

$$Yield\left(\frac{g_{AC}}{L_{Alpeorujo}}\right) = \frac{W_C}{V_O} = \frac{W_C}{\rho \cdot W_O} \cdot \frac{(100 - H)}{100} \quad (Eq. S2)$$

where W_C (g) is the dry weight of the prepared AC after purification, W_O (g), the dry weight of the *alpeorujo* before activation, V_O (L) the initial *alpeorujo* volume (20 ºC), H the moisture of *alpeorujo* (%) and ρ the density of *alpeorujo* (g/L).

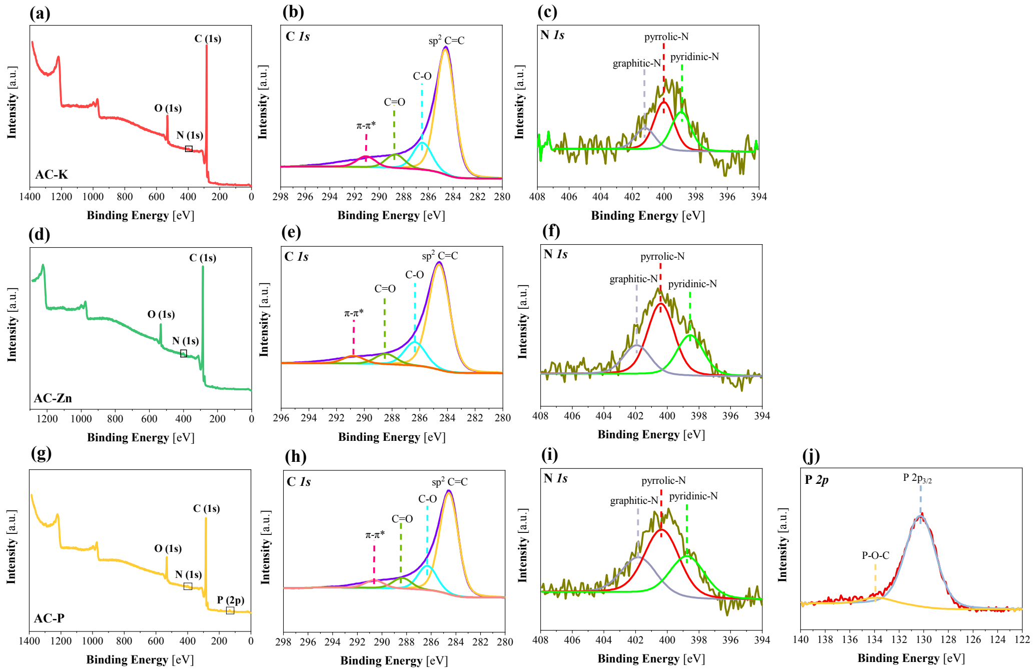

Figure S2. XPS spectra for **(a,b,c)** AC-K, **(d,e,f)** AC-Zn, and **(g, h, i, j)** AC-P samples.

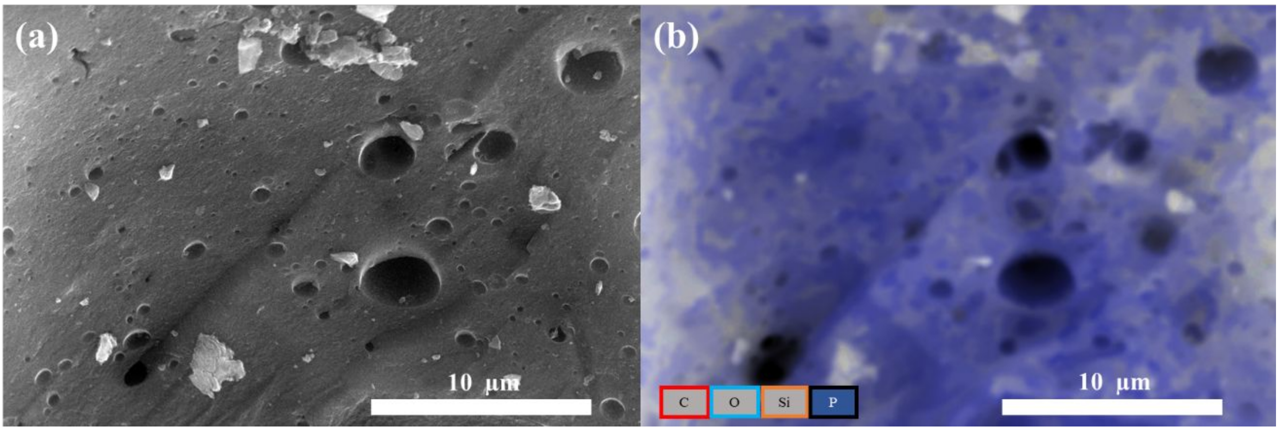

Figure S3. **(a)** SEM images and **(b)** EDS mapping of P for the AC-P sample.

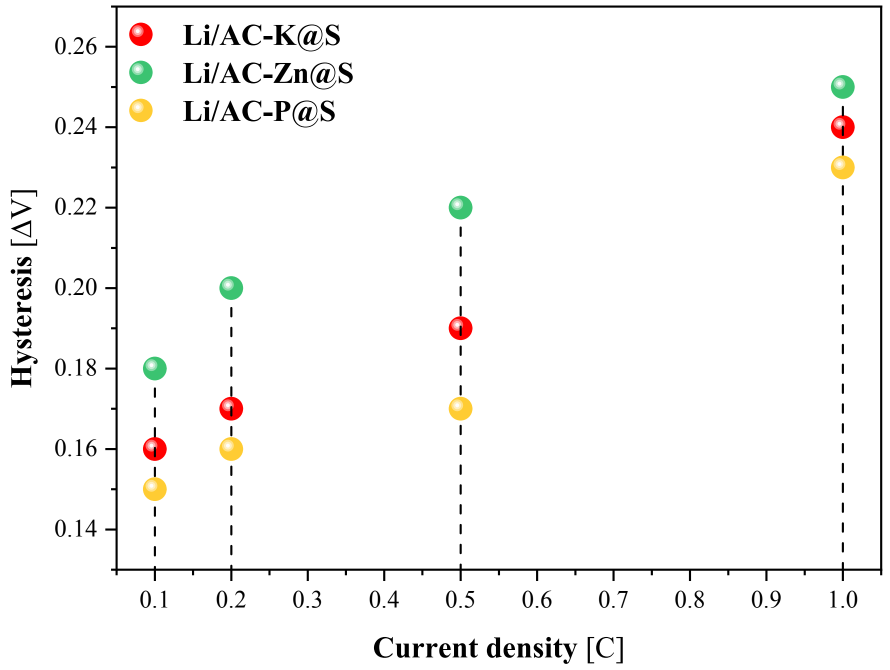

Figure S4. Hysteresis voltage calculated from the charges/discharges profile at different rates.

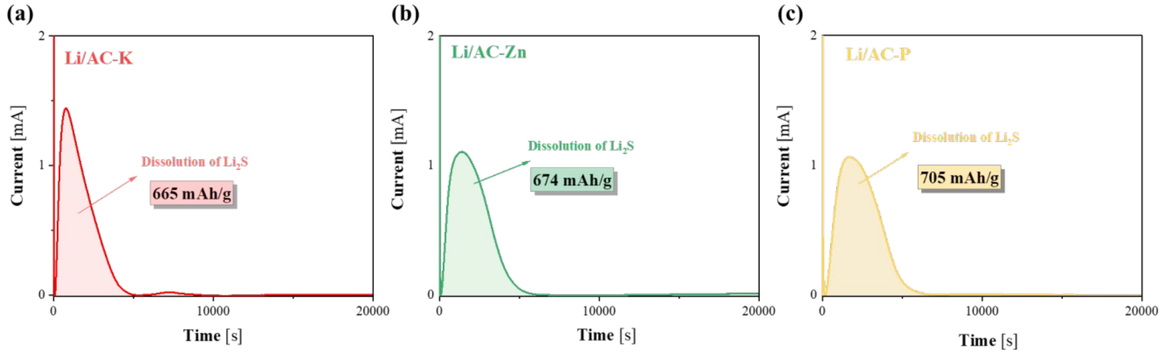

Figure S5. Potentiostatic charge curves of Li_2S dissolution **(a)** Li/AC-K, **(b)** Li/AC-Zn, and **(c)** Li/AC-P, respectively.

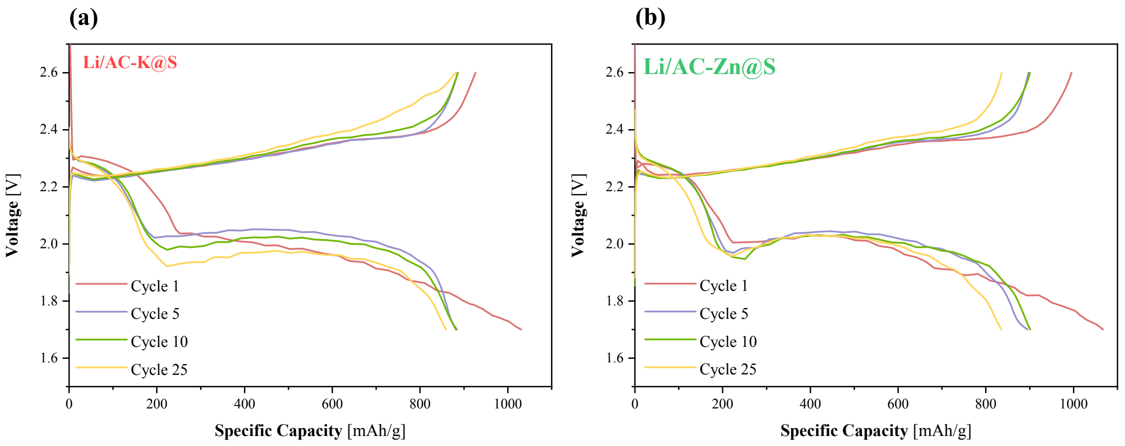

Figure S6. Charge–discharge profiles of the **(a)** Li/AC-K@S and **(b)** Li/AC-Zn@S electrochemical cell at 0.1C with S-loadings of 6.0 mg_S/cm^2.

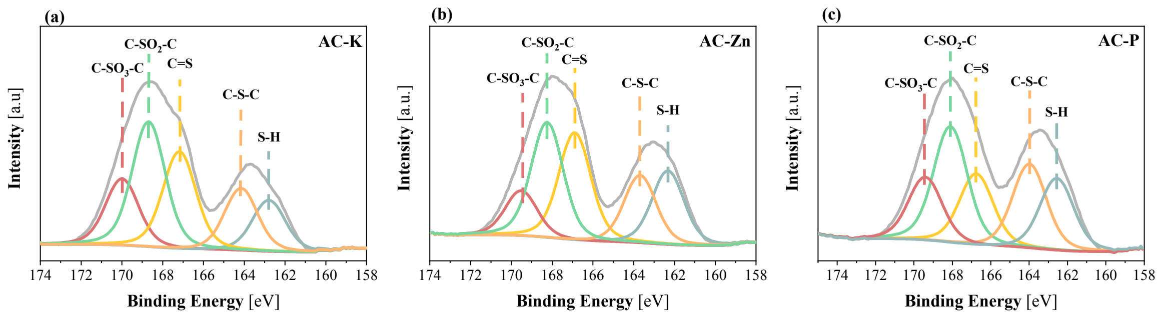

Figure S7. XPS spectra for the S *2p* bond region of samples **(a)** AC-K **(b)** AC-Zn, and **(c)** AC-P after the LiPS adsorption process.

Table S1. Physico-chemical characterisation of the *alpeorujo*.

Variables	*Values*	*Variables*	*Values*
Moisture (%)	70.02 ± 0.02	***P-P_2O_5 (mg/kg)***	2946 ± 10
pH	5.97 ± 0.02	***Ca (mg/kg)***	10,900 ±120
TC (mg/kg)	147,829 ± 15	***Mg (mg/kg)***	3640 ± 340
IC (mg/kg)	237 ± 5	***S (mg/kg)***	2090 ± 580
TOC (mg/kg)	147,592 ± 7	***Al (mg/kg)***	1320 ± 254
FS (mg/kg)	67,621 ± 1740	***Fe (mg/kg)***	721 ± 12
VS (mg/kg)	932,379 ± 2000	***Na (mg/kg)***	241 ± 18
N-TKN (mg/kg)	9816 ± 340	***Ni (mg/kg)***	38.7 ± 1.6

Table S2. Textural properties of AC-K, AC-Zn, and AC-P samples.

AC samples	S_{BET} *(m^2/g)*	S_{micro} *(m^2/g)*	V_T *(cm^3/g)*	V_{micro} *(cm^3/g)*
AC-K	1830	951	1.05	0.47
AC-Zn	579	411	0.34	0.20
AC-P	1058	626	0.68	0.31

Table S3. The results of the trapped sulfur in pores calculated theoretically and by TGA.

Sample	V_{micro} *(cm^3/g)*	$W_{S\ micro}$ *(%) - Theoretical*	W_S *(%) - TGA*	
			1st step	*2nd step*
AC-K	0.47	49.31	31.95	35.63
AC-Zn	0.20	29.28	63.13	7.70
AC-P	0.31	39.09	52.46	17.81

Table S4. Costs, hypothesis and results of the economic and energy balances for ACs and AC@S composites.

Cost	Electricity in Europe (industrial fee 2025)	€ 0.0564/kWh
	N_2	€ 0.5/m^3
	KOH	€ 790/t
	$ZnCl_2$	€ 1500 /t
	H_3PO_4	€ 900/t
	Sulfur	€ 500/t
	Alpeorujo	€ 0
Hypothesis	Mass ratio	2:1 (*Alpeorujo*:AA)
	Grinding (raw materials, d > 50 mm)	0.5 h, 100 W
	Drying stage	20-105 ºC
	Initial moisture	70 %
	Final moisture	10 %
	Oven power	2500 W
	Pyrolysis temperature	900 ºC
	Flowmeter power	2 W
	Grinding (AC@S 50 mm > d > 0.05 mm)	3h, 120 W
	Selected mass	1 $t_{wet\ alpeorujo}$
Results	Energy consumed (drying stage)	1829 MJ/$t_{wet\ alpeorujo}$
	Energy consumed (pyrolysis)	7942 MJ/$t_{wet\ alpeorujo}$
	Energy consumed (grinding)	400 MJ/ $t_{dry\ raw\ materials}$
	Energy consumed (grinding)	1296 MJ/$t_{dry\ AC@S}$
	**ACs estimated cost**	**€ 6.72 /kg_{AC-K}**<br>**€ 2.98 /kg_{AC-Zn}**<br>**€ 1.98 /kg_{AC-P}**
	**AC@S composites estimated cost**	**€ 2.41 /$kg_{AC-K@S}$**<br>**€ 1.29 /$kg_{AC-Zn@S}$**<br>**€ 0.99 /$kg_{AC-P@S}$**

Table S5. Li^+ ion diffusion coefficients (cm^2/s) of the Li/AC-K@S, Li/AC-Zn@S, and Li/AC-P@S cells calculated by applying the Randles-Sevcik equation.

Slope of the line				
	A_1	A_2	C_1	C_2
Li/AC-K@S	0.56	0.64	–0.49	–0.39
Li/AC-Zn@S	0.44	0.45	–0.17	–0.25
Li/AC-P@S	0.77	0.85	–0.37	–0.61
Diffusion coefficients (cm^2/s)				
	A_1	A_2	C_1	C_2
Li/AC-K@S	$9.66 \cdot 10^{-6}$	$1.27 \cdot 10^{-5}$	$7.53 \cdot 10^{-6}$	$4.78 \cdot 10^{-6}$
Li/AC-Zn@S	$6.11 \cdot 10^{-6}$	$6.44 \cdot 10^{-6}$	$8.51 \cdot 10^{-7}$	$1.94 \cdot 10^{-7}$
Li/AC-P@S	$1.84 \cdot 10^{-5}$	$2.26 \cdot 10^{-5}$	$4.23 \cdot 10^{-6}$	$1.17 \cdot 10^{-5}$

Table S6. The electrical conductivity results of different electrodes measured using the four-point probe test.

Electrodes	***Conductivity (S/cm)***
AC-K@S	100.15
AC-Zn@S	73.75
AC-P@S	120.63
